# Review of carbon dioxide removal and recycling in space

**DOI:** 10.1038/s41526-026-00608-x

**Published:** 2026-05-12

**Authors:** Cathryn O’Sullivan, Thomas M. Kohl, Kristina Konstas, Xavier Mulet, Kim Lee Chang, Matthew R. Hill, Anastasios Polyzos, Aaron W. Thornton

**Affiliations:** 1 Agriculture and Food Research Unit, Commonwealth Scientific and Industrial Research Organisation, St Lucia, QLD Australia; 2 Manufacturing Research Unit, Commonwealth Scientific and Industrial Research Organisation, Clayton, VIC Australia; 3 School of Science, RMIT University, Melbourne, VIC Australia; 4 Environment Research Unit, Commonwealth Scientific and Industrial Research Organisation, Hobart, TAS Australia; 5 Department of Materials Science and Engineering, Monash University, Clayton, VIC Australia; 6 School of Chemistry, Faculty of Science, University of Melbourne, Melbourne, VIC Australia; 7 Faculty of Science and Engineering, Southern Cross University, Lismore, NSW Australia

**Keywords:** Chemistry, Energy science and technology, Engineering, Environmental sciences

## Abstract

Recycling carbon dioxide (CO_2_) remains a challenge for long-duration crewed missions, as carbon is an essential element to produce vital resources. This article is a review of the state-of-the-art technologies for removing and recycling metabolically produced CO_2_ in microgravity environments, including stations, habitats, vehicles, and suits. The study covers the breathable air requirements for astronauts, supply requirements for missions, carbon removal technologies, and synthetic and biological conversion routes from CO_2_ to fuels, sugars, foods, pharmaceuticals, and materials.

## Introduction

In 2018, the United States announced plans to fund the Lunar Gateway project (officially Lunar Orbital Platform—Gateway), which is a proposed space station in lunar orbit as part of NASA’s Artemis programme. As of January 2024, international participants include the Canadian Space Agency (CSA), Japan Aerospace Exploration Agency (JAXA) and Mohammed Bin Rashid Space Centre (MBRSC). In parallel, India is planning a crewed mission to the Moon by 2040, China plans to establish a Lunar research station by 2035, and Russia plans to build a Moon base by 2031. The United States-led Gateway platform will serve as a hub for short-term habitation and deeper space exploration, including a crewed mission to Mars by 2040. The key challenges for deep space exploration were identified by NASA, including the recovery of O_2_ from CO_2_, long-duration food systems, and on-board manufacturing^1^. By creating a breathable atmosphere for astronauts and utilizing the exhaled waste products like CO_2_, longer missions can be supported.

Everyday items, including food, water and breathable air, are currently produced in transit or transported from Earth. For example, the International Space Station (ISS) requires deliveries 8–9 times per year that include fuel, food, clothing, medicine, furnishings, flight tools, cameras, parachutes, safety equipment, sleep restraints and research payloads^2^. Cargo costs can range from 10,000 to 40,000 USD per pound and rely on consistent, successful launches. In 2014, there were three different resupply missions that failed, resulting in 8 months without any supplies. For stations and habitats located further away from Earth, including the proposed Gateway and Lunar habitat, the challenges to resupply are amplified. In these scenarios, every molecule counts, and therefore, technologies to recycle components are vital. With a focus on CO_2_ and derivative products, this study reviews the technologies and opportunities of recycling breath into useful products.

**Fig. 1:**
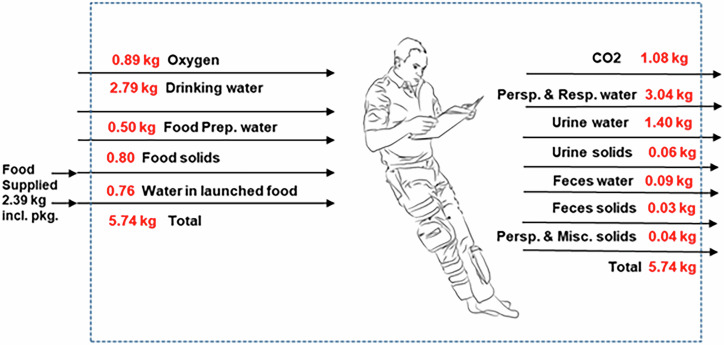
Daily mass balance of an 82 kg astronaut. This highlights the opportunity to recycle the carbon into useful products. All values are in units of kg. Reproduced with permission from Ewert and Stromgren^130^.

Figure 1 depicts a daily mass balance of an 82 kg astronaut, including oxygen, water and food, at a total of 5.74 kg. In addition, a mission requires medicines, fuels, clothing, tools, equipment and other materials, equating to ~30–50 kg of supplies per day per astronaut. Considering the output from respiration and perspiration (1 kg of carbon dioxide and 3 kg of water per day), there is an opportunity to recycle the carbon dioxide to minimize supplies from Earth. Noting there is a surplus of 0.48 kg of water that is produced metabolically by the astronaut. Between 2010 and 2018, the ISS utilized a Sabatier reactor to recover the oxygen O_2_, consequently dumping the remaining carbon in the form of methane (CH_4_). This process recycled around 50% of the CO_2_. Eventually, the Sabatier assembly was shut down due to degradation issues^3^. Given that carbon is an essential element for most products, alternative uses of CO_2_ and CH_4_ must be considered. For example, Jan has outlined potential options for CO_2_ recycling including^4^:

- CO_2_ for oxygen recovery using plasma pyrolysis or the Bosch reaction.
- CO_2_ for plant or algal growth.
- CO_2_ as a source of fuel production, including methane and hydrocarbon liquid fuels.
- CO_2_ as a feedstock (microbial substrates and fish food) for biomanufacturing.
- CO_2_ as a compressed gas for compressed gas cleaning or propulsion.
- CO_2_ as a carbon and oxygen feedstock for synthetic chemicals (plastics, pharmaceuticals, and adhesives).
- CO_2_ as a cleaning solution or switchable polarity solvent.

CO_2_ is one of the most important chemical feedstocks because it is non-toxic, non-flammable, abundant, economical, and intrinsically renewable. CO_2_ as a single carbon source has received considerable attention from the chemical community over the last several decades, since it could potentially replace toxic single carbon sources such as phosgene and derivatives. Particularly, the incorporation of CO_2_ into complex organic compounds has become an important topic in organic synthesis.

### Breathable air requirements

Maintaining breathable air is critical for long-term missions that depend on the health and fitness of the astronauts. Terrestrial air contains roughly 21% oxygen, 78% nitrogen, variable 0–3% water vapour, 1% argon, 0.04% carbon dioxide and small amounts of other gases. High levels of carbon dioxide are considered harmful, resulting in headaches, impaired cognition, drowsiness, increased heart rate, elevated blood pressure, and asphyxiation^5^. Many authorities have placed safety limits on the levels of exposure in workplaces on Earth, including the National Institute for Occupational Safety and Health (NIOSH), National Occupational Health and Safety Commission (NOHSC) of Australia, the American Society of Heating, Refrigerating and Air-conditioning Engineers (ASHRAE) and the World Health Organization (WHO). Overall, there is general agreement for an average 8-h exposure limit for partial pressure carbon dioxide (ppCO_2_) of 0.5% (or 3.8 mmHg) and a maximum limit of 4% (30.4 mmHg), which is considered dangerous to life and health^6^.

Since 2022, NASA’s average 1-h limit in a nominal vehicle or habitat has been lowered to 0.4% (3 mmHg) due to observed operational and research data. Prior to 2022, the requirements accepted a larger range of 0.5–1% (3.8–7.5 mmHg). For the ISS, the off-nominal ppCO_2_ level is 2% (15 mmHg), and the emergency ppCO_2_ level is 2.6% (20 mmHg). During the Apollo 13 mission, where the Lunar Module’s CO_2_ scrubber needed to support 3 astronauts instead of 2, the CO_2_ levels reached a maximum of 1.99% (14.9 mmHg)^7^.

In 2012, Cronyn et al. published a study reporting CO_2_ toxicity effects (headaches and lethargy) on astronauts that correlates with relative elevations of CO_2_^5^_._ Studies by Scully et al. agreed with these results to some extent, where decision-making and cognition performance declined when CO_2_ levels increased from a baseline of 0.04–0.12%^8^. However, cognitive performance returned to baseline results when CO_2_ levels increased further to 0.25% and beyond. It was suggested that the unique environment of space increases the sensitivity of astronauts to higher levels of CO_2_. Several factors were suggested to explain the unusual and unexpected findings, including the amount of sleep the participants had before the study, differences in characteristics across astronaut-like personnel and submariners, and age differences between the populations. Nonetheless, there are severe health outcomes at high concentrations 0.65–1.85% or prolonged exposure over a few days^9^.

Considering that humans exhale 4% carbon dioxide at a rate of 1 kg per day and that the exposure limits should be below 0.4%, this presents a great scientific challenge. The lower the levels of CO_2_ in the air, the more difficult it is to capture. This partly explains why lower CO_2_ levels in space have not been achieved.

Optimum humidity levels are between 40% and 60% relative humidity (RH) (1–1.5 mol%), with safe limits between 30% and 70% RH (0.7–1.7 mol%). Levels below 30% RH can cause respiratory conditions such as sinusitis and dry eyes, while levels above 70% RH can cause discomfort, including excessive perspiration, tiredness, breathing difficulty and loss of temperature control within the body.

In addition to carbon dioxide and water, there are contaminants that need to be limited within the cabin environment^10^. NASA has reported over 200 contaminants that are a result of equipment off-gassing, human metabolism and cleaning fluids during pre-flight ground processing. Many factors contribute to trace chemical contamination, including the vehicle configuration, crew size and activities, mission duration and materials selection. According to the Russian air quality standards, contaminants like ethanol should be kept below a concentration of 10 mg/m^3^ (or 1%).

For the International Space Station, it has been suggested in a presentation by Jan^4^, that the carbon dioxide removal system should meet the following criteria:

- Mass—450 pounds (181 kg)
- Power—1000 W
- Volume—19 cubic feet (538 L)
- Heat rejection—620 W to thermal control system (TCS) and 500 W to Avionics Air
- CO_2_ removal performance—4.16 kg/day, assuming crew of 4
- CO_2_ concentrations in cabin—2.0 mmHg (2632 ppm or 0.26%)
- Air flow—26 CFM (736 LPM)

### Supply requirements

In 2020, the Russian Progress cargo ship delivered 2.8 tons of supplies and equipment to the International Space Station. As an example of the supply requirements for space travel, this cargo ship delivered 772 kg of propellant, 50 kg of oxygen, 417 kg of water and 1347 kg of dry cargo, including food and equipment. Given that supplies like these are delivered 8–9 times per year, it is worth understanding the molecular composition of the supplies and exploring whether these supplies can be recycled or manufactured from chemical building blocks such as carbon dioxide.

#### Propellants

The ISS requires an average of 7 tons of propellant each year for altitude maintenance, debris avoidance and attitude control^11^. Two pairs of 200 L propellant tanks (two nitrogen tetroxide N_2_O_4_ and two unsymmetrical dimethyl hydrazine UDMH) provide a total of 860 kg of usage propellant^12^. The propulsion system rocket engines use the hypergolic reaction, i.e., spontaneous combustion upon contact.

For launch capability from Earth, SpaceX Draco rocket engines use a combination of nitrogen tetroxide N_2_O_4_ and monomethyl hydrazine that also spontaneously combust upon contact. McCormack has published a list of propellants available^13^. The main criteria for a good propellant include a large heat of combustion and the production of products containing simple, light molecules embodying such elements as hydrogen (the lightest), carbon, oxygen, fluorine, and the lighter metals (aluminium, beryllium and lithium).

For long-term missions to the Moon and Mars, propellants will play a critical role in transportation between habitats. SpaceX has proposed to mine the CO_2_ from the atmosphere of Mars in combination with water from the rocks, and generate methane and oxygen as propellants to support interplanetary transport. Launching from Earth requires a load of fuel because of the strong gravitational pull. For example, the Soyuz uses around 150 tons of fuel for each launch. The Moon has one-sixth the gravity of Earth and is therefore an attractive base for launching to Mars and beyond. Ideally, the propellants would be manufactured on the Lunar surface to avoid the additional costs of transporting from Earth. The most straightforward method is to convert the water from the ice reserves to hydrogen and oxygen using hydrolysis, powered by solar panels.

#### Oxygen

Oxygen is currently delivered to the space station to replenish the atmosphere whenever the ISS receives a supply shuttle in the order of 50–100 kg per trip. The average person needs around 0.84 kg of oxygen per day to survive. The NASA Oxygen Generation System and the Russian Elektron system generate around 2 kg of oxygen per day. It uses water from the Water Reclamation System and electrolysis to generate oxygen and hydrogen. Up to 2017, the hydrogen was fed into the Sabatier System to convert carbon dioxide into methane and water (CO_2_ + 4H_2_ → CH_4_ + 2H_2_O + energy). The total Gibbs free energy is approximately −113.5 to −130.8 kJ/mol at 298 K, making the reaction highly spontaneous and favourable for producing methane at low to moderate temperatures. Unfortunately, the Sabatier catalyst was contaminated and subsequent operation ceased^3^. This means that the captured CO_2_ is currently vented overboard, hence the station loses valuable elemental carbon and oxygen. If the Sabatier was reinstalled, the process would result in a loss of elemental carbon C and hydrogen H that would be vented overboard in the form of methane (CH_4_).

#### Water

As shown in Fig. 1, astronauts require around 5 kg of water per day for consumption^14^. Approximately 98% of the water is recycled using the Environmental Control and Life Support System (ECLSS), which includes the Common Cabin Air Assembly to condense water vapour from air^15^. Water supplies are supplemented by regular deliveries on the order of 400 kg per cargo ship. Considering that both the Moon and Mars have water resources, there may be no need to transport water from Earth to these destinations. The travel time to Mars of ~500 days will require significant amounts of water, and the mining technology and energy costs to melt and process frozen water are unknown.

#### Food and nutrition

Astronauts on the ISS currently rely on food deliveries from Earth for all their nutritional needs. For long-term missions, astronauts will need to become more independent with less reliance on deliveries from Earth. The nutritional requirements are guided by the Food Guide Pyramid from the U.S. Department of Agriculture^16^. A healthy diet includes, on average, 40% bread, cereal, rice & pasta, 19% vegetables, 15% fruit, 11% meat, poultry, fish, dry beans, eggs & nuts, 11% milk, yogurt & cheese, and less than 4% fats, oils and sweets. Every mission must ensure a secure and reliable supply of these food groups.

The baseline food and beverage list for the space shuttle provides an example of the “shopping list” for a space mission^16^. The list includes a variety of items to meet the requirements, including beef, bread, sweets, cereals, cheese, chicken, eggs, fruit, ham, noodles, potatoes, salmon, soups, tuna, turkey, vegetables, fruit juices, coffee and condiments.

**Fig. 2:**
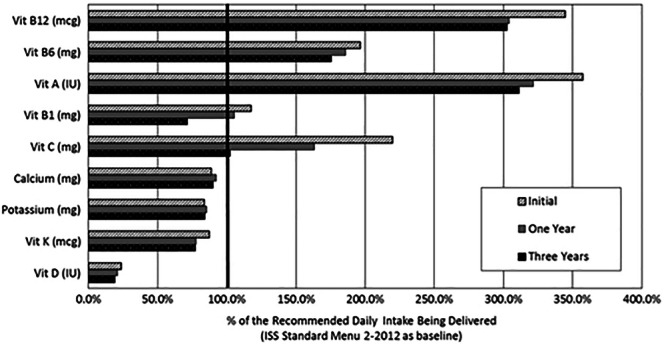
Expected vitamin delivery for aging space diet consumed according to the ISS Standard Menu. Reproduced with permission from Cooper et al.^17^.

Cooper et al. assessed the nutritional quality of space food over three years (see Fig. 2)^17^. The results indicated that the current food may not have adequate concentrations of potassium, calcium, vitamin D and vitamin K, to meet the recommended daily intake even before storage. Furthermore, the study observed degradation of vitamins A, C, B1, and B6 over the storage period of 3 years. This led to the conclusion that the food supplies need more nutrients and better storage methods to preserve those nutrients.

#### Medicines and other materials

The ISS has several medical kits containing medicine and equipment from day-to-day conveniences to emergency procedures^18^. For example, the contents within the Convenience Medication Pack include antibiotics, antidiarrheals, antihistamines, decongestants, lubricants, pain relievers, sleep aids, steroids, stimulants and stool softeners. Medicines have a shelf life and need to be replaced on a regular basis. In addition to regular terrestrial degradation of medicines, there have been several studies confirming that some medications degrade faster in space, summarized by Blue et al.^19^. The medications found to be unstable after spaceflight include ibuprofen, melatonin and sertraline.

Given the vast number of medicines and medical equipment that are required for space missions, there is an opportunity to manufacture some of these products in space. NASA has highlighted this opportunity through their CO_2_ Conversion Challenge to “convert CO_2_ into molecules to power bio-manufacturing in space”.

Mane reported a list of materials required for space missions, including clothing, paper & office supplies, wipes/tissues, towels, foam packaging, tools, EVA supplies and others^20^. This results in a total mass of around 1.5 kg per day per crew member. Considering a Gateway mission and a Mars mission, this leads to a total of 1–2 tons of materials required.

## State-of-the-art technologies

### Carbon dioxide removal from the atmosphere

Carbon dioxide removal is necessary for any confined environment where humans are present. Humans exhale around 1 kg of CO_2_ per day and levels should remain below 0.4%. Carbon removal technologies have been developed for submarines, deep sea divers, underground mining facilities, space shuttles, space stations and space suits. The type of technology adopted depends on the volume, energy and urgency requirements of the application. An alternative to carbon dioxide removal is the continuous supply of pure oxygen or air via compressed vessels such as those carried aboard Vostkov-1 in 1961, while excess air is vented overboard. However, this approach is only practical for short-duration missions, as the mass and cost become prohibitive for long-duration operations.

For space applications, a review of technologies past, present and future was provided by Honeywell Aerospace^21^. A history of carbon removal technologies for U.S. space vehicles, including Mercury, Gemini, Apollo, Skylab, Shuttle and International Space Station, is summarized in Table 1. A description of the state-of-the-art technologies is provided in the following subsections.

**Table 1:** Carbon dioxide removal technologies used in space^21^

Technology	Space vehicle	Developer	Period	No. of missions
Lithium Hydroxide	Mercury	Honeywell	1958–1963	6
Lithium Hydroxide	Gemini	Honeywell	1962–1966	10
Lithium Hydroxide	Apollo	Honeywell	1961–1972	10
Lithium Hydroxide	Space Shuttle	Hamilton-Sundstrand	1985–2011	5 shuttles & 135 launches
Zeolite Molecular Sieve	Apollo	Honeywell	1965–1979	Zero
Zeolite Molecular Sieve	Skylab	Honeywell	1973–1979	3
Zeolite Molecular Sieve	ISS	Honeywell	2002–present	Manned for 21 years
Amine Solid Sorbent	Mir Space Station	Zvezda	1986–2001	Manned for 10 years

#### 4-Bed molecular sieve (4BMS)

**Fig. 3:**
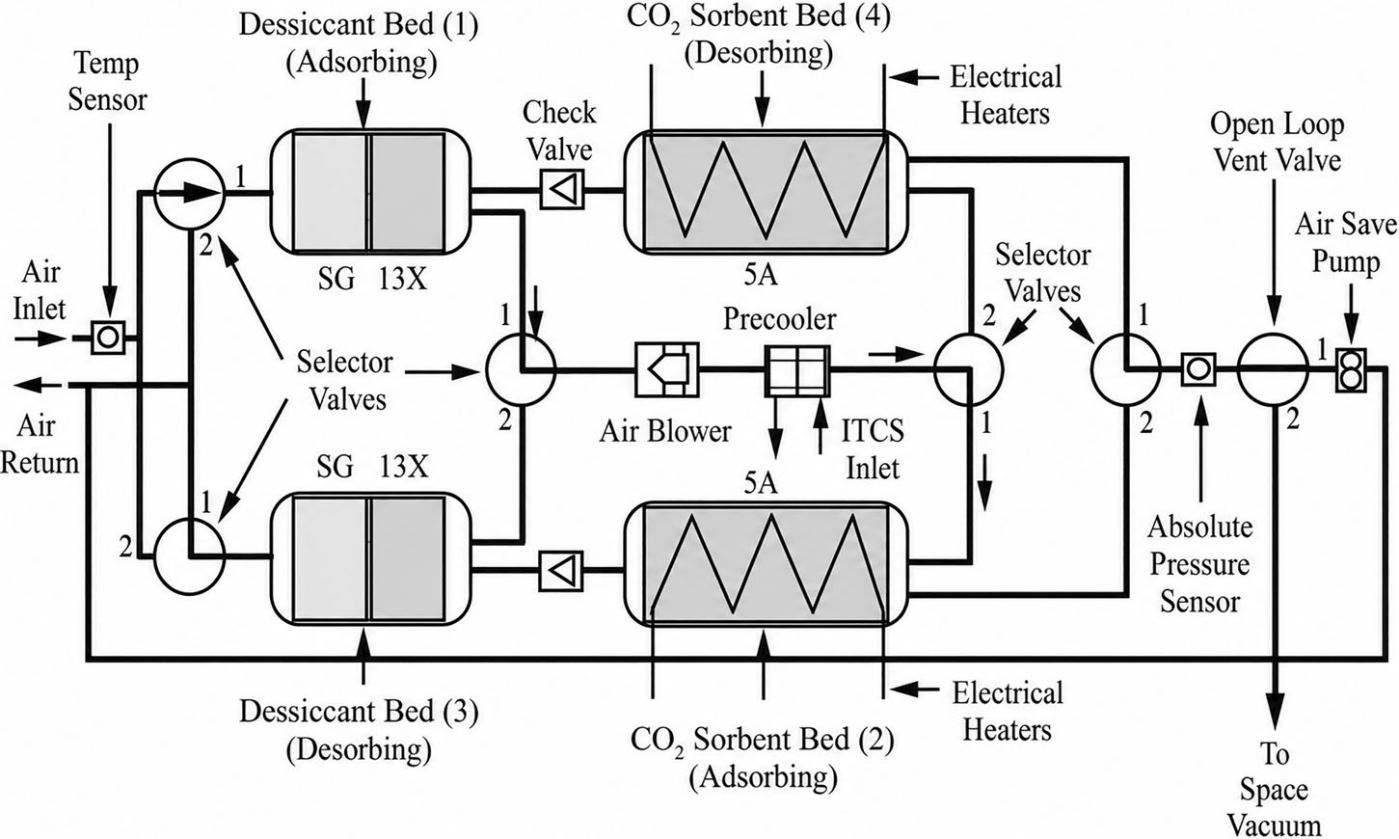
Schematic of the 4-bed molecular sieve (4BMS) unit within carbon dioxide removal assembly (CDRA) showing two desiccant beds, two CO_2_ sorbent beds, air blower, pre-cooler, electric heaters, valves, and air save pump. Reproduced with permission from Knox et al.^22^.

**Fig. 4:**
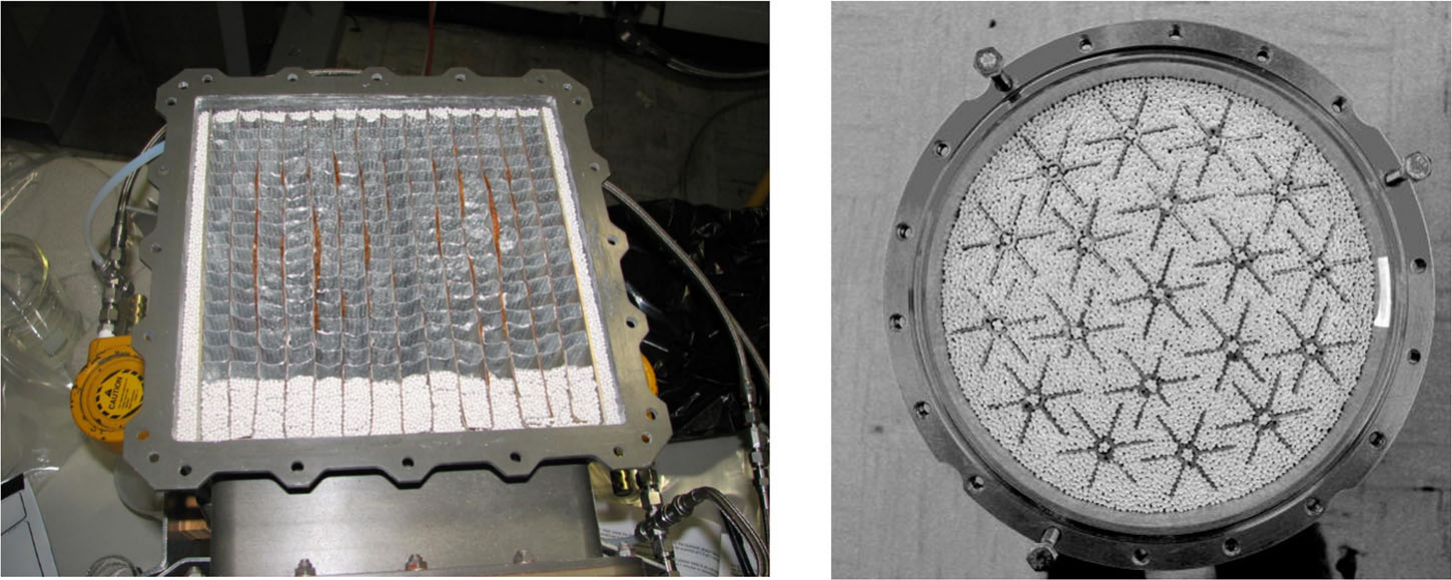
Transition from a box-shaped housing for CDRA (left) to a cylindrical vessel for the 4BCO_2_ Scrubber (right). The CDRA vessel is packed with zeolite 5A beads, and the 4BCO_2_ Scrubber vessel is packed with zeolite 13X beads. The zeolite beads are surrounded by electrical heaters to accelerate the desorption under vacuum. Left picture reproduced with permission from Coker et al.^131^. Right picture reproduced with permission from Cmarik et al.^23^.

The ISS has used the 4-Bed Molecular Sieve (4BMS) technology since the beginning, integrated within the carbon dioxide removal assembly (CDRA). The 4BMS combines two desiccant beds for removing moisture and two CO_2_ sorbent beds for removing carbon dioxide, see Fig. 3^22^. The desiccant beds are typically packed with silica gel, while the CO_2_ sorbent beds contain zeolite. In 2021, the 4BMS system was upgraded to the 4-Bed CO₂ Scrubber (4BCO₂). This updated design introduced several improvements, including a transition from a box-shaped housing to a cylindrical vessel to provide more uniform airflow and heating, shown in Fig. 4. Additional enhancements included magnetic-bearing air blowers to minimize mechanical wear, a redesigned heater core for improved thermal distribution, more robust valve designs to reduce sorbent dusting, and shorter operating cycles with modified zeolite layering to prevent condensation^23–26^.

Optimization over the years has resulted in a desiccant bed containing silica gel and zeolite 13X, and CO_2_ removal bed containing zeolite 13X beads^27^. The desiccant beds are required because the CO_2_ capacity of the zeolite is significantly reduced in the presence of water due to competitive adsorption. The captured water is released back into the cabin after each cycle. The process is a temperature-vacuum swing adsorption process combining electrical heating within the bed with vacuum to accelerate the desorption phase. Recent modifications include the Sabatier unit shown in Fig. 3 that converts the captured CO_2_ into water and methane. The water is recycled while the methane is vented overboard.

One of the downfalls of this technology is the degradation of the beads over time into particles that block the bed and can also be exhausted into the cabin. For this reason, a large range of sorbents has been explored to minimize attrition. For example, Knox et al. compared candidates and their stability at a range of humidities, characterized by dew point^28^. In this experiment, Grade 564 3A zeolite was the most stable, but other experiments, such as CO_2_ capacity and crush testing, indicated that Grade 544 13X and BASF 13X proved the best overall performance.

Although zeolites exhibit lower CO_2_ capacity in the presence of moisture, various strategies have been employed to improve and adapt their performance in different environments. For example, zeolites have been made more selective for CO_2_ over water, consequently removing the need for a silica gel, which would reduce the size and weight of the 4BMS unit. Specifically, zeolite Na-SAPO-34, which is highly hydrophilic, was exchanged with Strontium ions to give Sr-SAPO-34, achieving a CO_2_ working capacity of 1.70 wt% in 10% relative humidity (RH) and 1.22 wt% CO_2_ in 50% RH^29^. Although the CO_2_/H_2_O selectivity was not reported for the modified Sr-SAPO-34, the results indicate a 30% reduction in CO_2_ capacity between 10% and 50% RH. In comparison, other studies have shown that Na-SAPO-34 loses 60% of CO_2_ capacity under the same change of conditions, and 96% reduction of CO_2_ capacity compared with dry conditions^30,31^.

Zeolites can simultaneously remove water vapour and CO₂ in an open-loop system where both are vented rather than recovered. For instance, the sorbent-based atmosphere revitalization (SBAR) system is being considered for lunar habitats, where pressure-swing adsorption (PSA) can operate for extended periods before thermal regeneration is required. Although water is not recovered in this approach, unlike on the ISS, the system reduces reliance on consumables compared with non-regenerable LiOH-based systems^32^.

Despite the reduced working capacity in higher humidity conditions, there is significant progress in improving zeolite’s ability to capture CO_2_ in lower partial pressures. For example, Jayaraman et al. presented CO_2_ adsorption isotherms on the Sr-SAPO-34 and Na-SAPO-34 zeolites at 30 °C. The results indicated that the strontium exchanged material achieves an increase in CO_2_ capacity at low partial pressure (<5 mmHg)^29^.

#### Lithium hydroxide (LiOH) cartridge

**Fig. 5:**
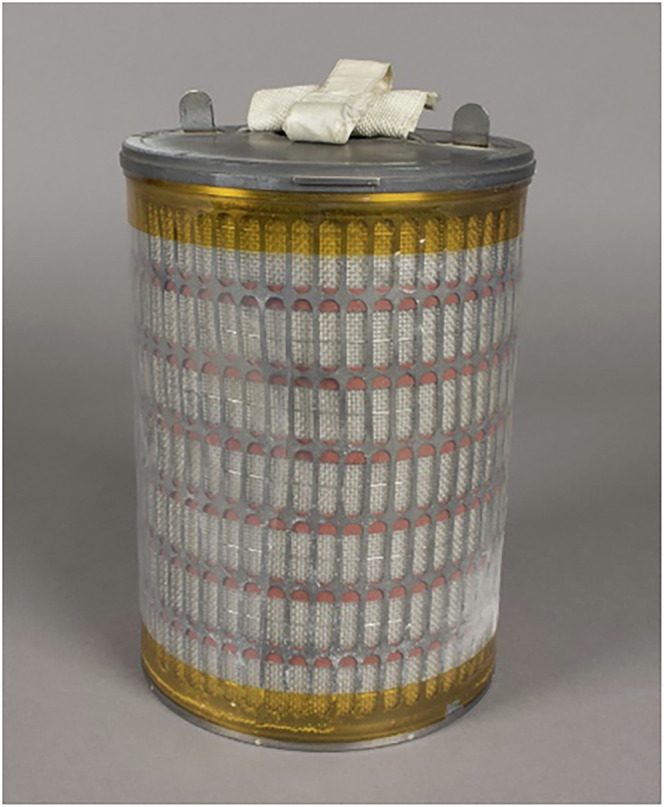
Lithium Hydroxide (LiOH) cartridge used as a backup system for eliminating excess carbon dioxide. Photo reproduced with permission from Mock-Up of canister from Apollo 13 Emergency, displayed at the Smithsonian National Air and Space Museum.

Lithium hydroxide (LiOH) was used on the Apollo 13 mission in 1970 as a non-regenerable technology to remove excess carbon dioxide. During this mission, an explosion in the Service Module forced the astronauts to shut down the Command Module and move into the Lunar Module as a lifeboat. The Lunar Module depended on two LiOH filters to remove excess, but was only designed for two astronauts instead of three. Engineers at Mission Control worked out a way to disassemble the spare LiOH filters from the Command Module and retrofit them to the Lunar Module to handle the additional carbon dioxide. Now it is used on the ISS as an emergency backup system (see Fig. 5).

The chemical reaction of LiOH with carbon dioxide is as follows,

$$2\,{\rm{LiOH}}\left({\rm{s}}\right)+2\,{{\rm{H}}}_{2}{\rm{O}}\left({\rm{g}}\right)\to 2\,{\rm{LiOH\cdot }}{{\rm{H}}}_{2}{\rm{O}}({\rm{s}})$$

$$2\,{\rm{LiOH\cdot }}{{\rm{H}}}_{2}{\rm{O}}({\rm{s}})+{\rm{C}}{{\rm{O}}}_{2}({\rm{g}})\to {{\rm{Li}}}_{2}{{\rm{CO}}}_{3}({\rm{s}})+3\,{{\rm{H}}}_{2}{\rm{O}}({\rm{g}})$$

with the net reaction as

$$2\,{\rm{LiOH}}({\rm{s}})+{{\rm{CO}}}_{2}({\rm{g}})\to {{\rm{Li}}}_{2}{{\rm{CO}}}_{3}({\rm{s}})+{{\rm{H}}}_{2}{\rm{O}}({\rm{g}})$$

where 2 Lithium hydroxide ions react with one carbon dioxide module to form one lithium carbonate salt and one water molecule in the presence of moisture^33^. The heat of reaction is around 90 kJ/mol-CO_2_. The theoretical binding capacity of LiOH is 0.92 kg-CO_2_/kg-LiOH based on the stoichiometric limit of the reaction^34^. Experimental capacity is observed to be around 0.5 m^3^/kg-LiOH and is manufactured as a solid powder, which is formed into structures such as pellets, rods or candles. The sorbent is generally not thermally regenerable as it forms lithium oxide under high temperatures.

#### Solid amine CO_2_ removal systems

**Fig. 6:**
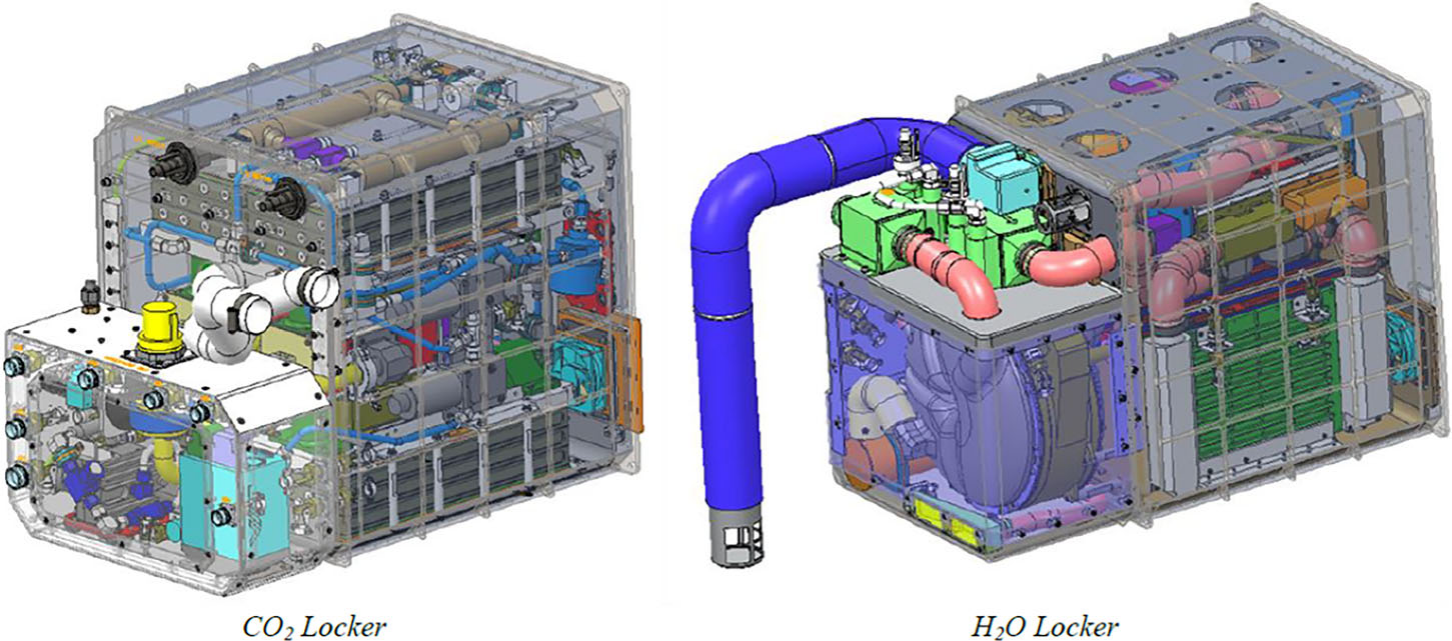
CAD models of the thermal amine scrubber (TAS) CO_2_ and H_2_O lockers. Reproduced with permission from Ranz et al.^35^.

In 2017, Ranz et al. from Collins Aerospace designed and tested a thermal amine scrubber (TAS) under a contract with NASA^35–38^. The prototype was based on a thermally regenerated solid amine adsorbent and was designed to remove 3.7 kg per day of CO_2_ at 2 mmHg partial pressure (2632 ppm). Two double lockers within an ISS Express Rack contained the Water Save subsystem (H_2_O locker), a CO_2_ Removal subsystem (CO_2_ Locker), and an ISIS drawer for the system controller (see Fig. 6). Similar systems using solid amine adsorbents include the CO_2_ And Moisture Removal Amine Swing-bed (CAMRAS)^39^ and the rapid cycle amine (RCA)^40^. In 2014, CAMRAS was being designed for the new Orion vehicle (a partially reusable space capsule for NASA’s human spaceflight programmes) based on tests conducted aboard the ISS between 2013 and 2014. Orion now utilizes the CAMRAS technology within the CO_2_ and Humidity Control (CHC) system^41^.

The RCA system has been designed as a portable life support system (PLSS) designed to allow an astronaut to conduct an extravehicular activity (EVA) or spacewalk. Three prototypical RCA units have been designed, built and tested based on a proprietary sorbent called SA9T. The SA9T can adsorb both CO_2_ and H_2_O vapour and desorbs under vacuum conditions. The two beds are in thermal contact, which allows the heat from adsorption to be redirected and used during the desorption phase.

In comparison with zeolites, solid amine systems generally require less energy to regenerate, as zeolites require >200 °C, while solid amines can be regenerated at <70 °C. For example, TAS was shown to require <1 kW of power while the 4BMS requires 1–1.5 kW^35^. In 2014, Button and Sweterlitsch reported slight degradation of the CAMRAS system over the course of 1000 h^39^. In 2024, Ranz et al. reported negligible degradation of the TAS system over multiple years of operation^38^.

#### Metal oxide (METOX) canisters

**Fig. 7:**
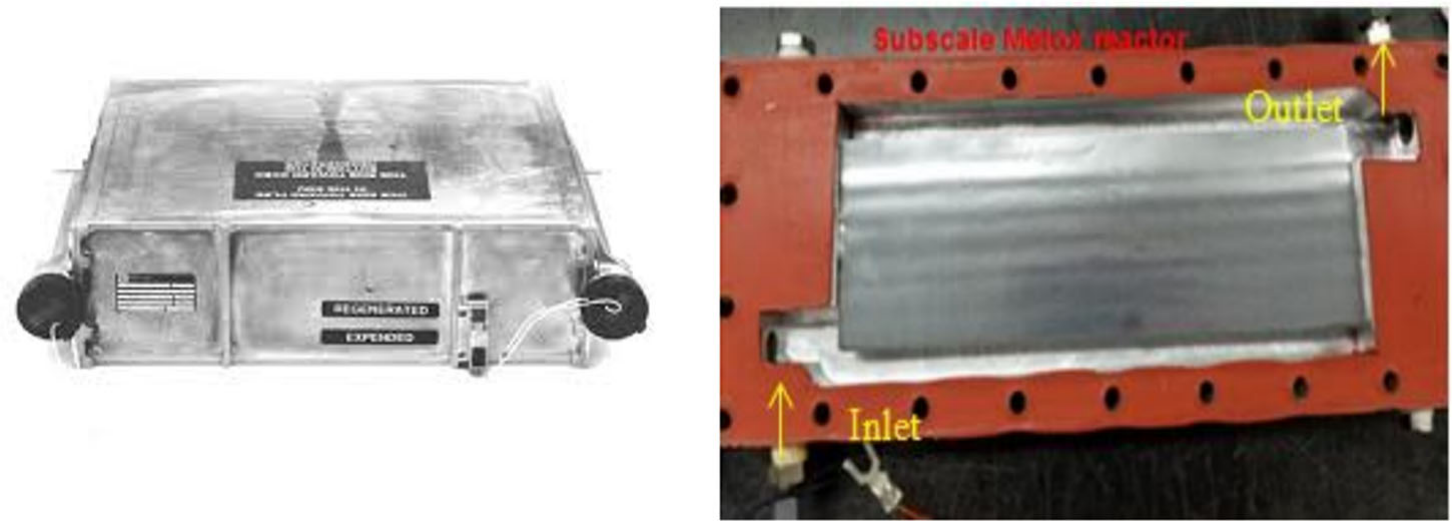
Metal oxide (METOC) canister containing stacked sheets of silver oxide. Reproduced with permission from Auman Jr et al.^42^.

Collins Aerospace originally developed the metal oxide (METOX) carbon dioxide scrubber (Fig. 7) in the 1990s to replace the non-regenerable LiOH canisters for NASA’s Extravehicular Mobility Unit (EMU). This technology remains the main CO_2_ scrubber for extravehicular activity. It utilized a silver oxide sorbent that reacts with carbon dioxide to form silver carbonate, which is thermally regenerable. The silver oxide is manufactured as a paste, which is formed into rectangular sheets and wrapped in a microporous polytetrafluoroethylene (PTFE) film^42^.

Thermal regeneration is performed on orbit by flowing heated air at 150–205 °C from the ISS cabin for 10 h. Some degradation has been observed. For example, the canister was cycled for 100 cycles and the observed degradation in performance was ~7.5%.

A range of other metal oxides, including zinc oxide, magnesium oxide and calcium oxide, have promising capture properties. Studies on 14 different metal oxides for CO_2_ capture, indicated a range of capacities from 20 to 150 wt% and isosteric heat of adsorptions from −40 to −400 kJ/mol-CO_2_^43^. MgO, for example, has a high theoretical capture capacity of 1100 mg CO_2_ /g-sorbent and can be regenerated at temperatures of 500 °C^44^.

#### Carbon dioxide removal by ionic liquid sorbent (CDRILS) systems

**Fig. 8:**
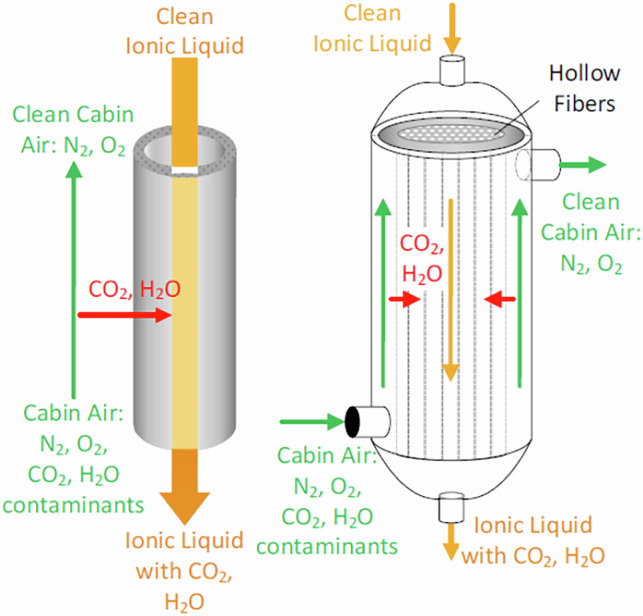
Individual hollow fibre (left) and membrane contactor (right) in scrubber configuration within the CDRILS system. Figure reproduced from Yates et al.^45^ with permission of Honeywell International Inc. Copyright © 2019 Honeywell International Inc. All Rights Reserved.

A collaboration between Honeywell International, NASA, the University of Alabama and Jacobs Engineering resulted in the scale-up and testing of the carbon dioxide removal by ionic liquid sorbent (CDRILS) system^45^. The technology is based on an ionic liquid confined within a hollow fibre membrane that allows the air to contact the liquid in a micro-gravity environment (see Fig. 8). It is claimed to be simpler than solid sorbent technologies because there is no need to switch between adsorption and desorption. The ability to rapidly flow the selective liquid through the air also allows the reduction of system size compared with solid sorbents. The ionic liquids are non-flammable, non-toxic, easily handled liquids with viscosities low enough for rapid mass transfer and fluid flow. For the latest design, 1-ethyl-3-methylimidazlium acetate (EMIM Ac) was the ionic liquid of choice for scale-up and testing.

Chemical degradation into impurities of the liquid was observed under 90 °C, which indicates the importance of avoiding high temperatures. Stripping CO_2_ from the ionic liquid requires heating to 50–60 °C. This is accomplished by passing the liquid through a heater and then through a hollow fibre stripping column where a vacuum pulls the desorbed CO_2_ from the fibre. Minor degradation of the hollow fibres was also observed after 8 months of operation.

#### Soda lime

Soda lime is a widely available sorbent sold in granular form for closed environments such as general anaesthesia, submarines, rebreather and recompression chambers. CO_2_ capacity of soda lime is around 26 L/100 g sorbent^46^ with a heat of reaction around 13.7 kcal/mol^47^. It is made from slaked lime with concentrated sodium hydroxide solution. The composition is 75% calcium hydroxide, Ca(OH)_2_, 20% water, H_2_O, 3% sodium hydroxide, NaOH and 1% potassium hydroxide, KOH. Manufacturers add a dye that changes colour indicating when the soda lime has reached full capacity of CO_2_. Regeneration is possible but at high temperatures of 1000 °C at which point the calcium carbonate CaCO_3_ (chalk) is converted to calcium oxide CaCO (lime) which forms calcium hydroxide Ca(OH)_2_ when slaked with water.

#### Cryogenic separation using super-cooled fin contactors

**Fig. 9:**
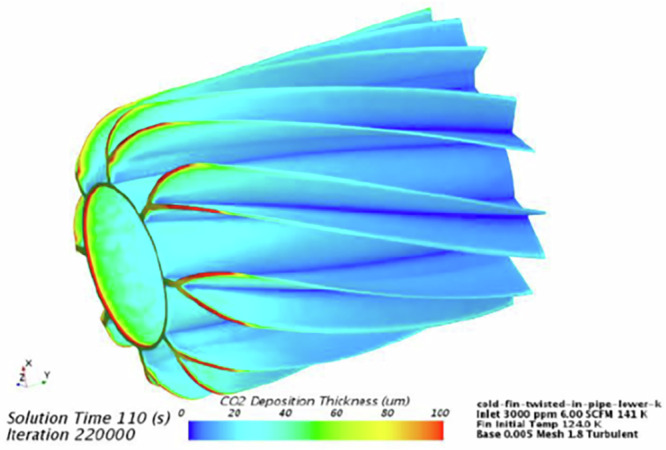
CO_2_ ice thickness on the cold surface after 110 s. Inlet conditions are 6 SCFM, 3000 ppm CO_2_, inlet air at 141 K, cold tip at 114 K and finhead at 124 K. Reproduced with permission from Belancik et al.^48^.

Belancik et al. proposed and tested a method to freeze CO_2_ out of the air using a super-cooled fin down to 114 K^48^. The method leverages the phase change temperature of air constituents to selectively remove CO_2_. Advantages of this method include the simplicity with minimal components and the avoidance of handling media such as sorbents, liquids and chemicals. One of the main disadvantages is the energy cost to cool both the incoming air and the contactor. The testing proved that the method was capable of continuously removing CO_2_ from the air (at 3000 ppm) (see Fig. 9). However, the method was not capable of capturing the target scale of one crew member, i.e., 1 kg per day.

#### Emerging materials for CO_2_ removal

Recent research has highlighted the potential of advanced porous materials for carbon dioxide removal in spacecraft life-support systems. In particular, metal-organic frameworks (MOFs) and functionalised sorbents have attracted significant attention due to their highly tunable structures and exceptional adsorption properties^49,50^.

These materials offer several advantages compared with traditional sorbents, including: (1) Extremely high surface areas, which provide a large number of adsorption sites for CO₂ molecules. (2) Tunable pore size and surface chemistry, enabling selective adsorption and optimization for specific operating conditions. (3) Improved adsorption capacity at low CO₂ concentrations, which is particularly important for maintaining safe atmospheric levels in crewed spacecraft.

Because of these characteristics, advanced porous materials are considered promising candidates for next-generation CO₂ capture systems in space habitats. Current research focuses on improving adsorption capacity, reducing regeneration energy requirements, and enhancing long-term durability under repeated adsorption–desorption cycles typical of spacecraft environmental control systems.

Research into advanced sorbent materials for spacecraft environmental control systems has explored a range of technologies capable of improving carbon dioxide removal and air revitalization performance. Harris et al. evaluated several solid-state sorbents for potential use in space life-support systems, assessing their adsorption capacity, regeneration behaviour, and stability under operating conditions relevant to spacecraft atmospheres^51^. The study identified key performance metrics required for long-duration missions, including durability over repeated adsorption–desorption cycles and efficient regeneration using limited spacecraft power resources. Richardson et al. investigated multifunctional sorbent (MultiSORB) devices designed to simultaneously remove carbon dioxide and trace contaminants from spacecraft cabin air. This work demonstrated that integrating multiple sorption functions within a single device could reduce system mass and complexity while maintaining effective atmospheric purification^52^.

Earlier work by Wójtowicz et al. examined carbon-based regenerable sorbents capable of removing both CO₂ and trace contaminants, highlighting the potential of engineered carbon materials with high surface area and tailored pore structures for multifunctional air-revitalization systems^53^. These materials showed promise for improving sorption performance while maintaining long operational lifetimes in cyclic systems. Knox et al. further investigated the role of desiccants and CO₂ sorbents in environmental control and life-support systems, analysing the interaction between humidity removal and carbon dioxide capture in adsorption beds. Their results emphasized the importance of integrated sorbent design to ensure stable performance in the humid cabin environments typical of crewed spacecraft^28^.

Finally, Thornton et al. explored the development of metal-organic frameworks (MOFs) as high-efficiency carbon capture materials, demonstrating how their tunable pore structures and high surface areas enable enhanced CO₂ adsorption at low concentrations. Together, these studies illustrate the continuing evolution of sorbent materials and system architectures aimed at improving the efficiency, durability, and integration of carbon dioxide removal technologies for future space missions^54,55^.

### Synthetic conversion of carbon dioxide

Synthetic conversion of carbon dioxide is necessary to recycle the captured carbon and oxygen elements. The Sabatier reaction was used on the ISS to recover the oxygen; however, the carbon is lost due to safety concerns of handling the methane by-product. Alternative synthetic routes are discussed, which open up potential opportunities to convert carbon dioxide into a range of useful products, including methanol, formaldehyde, formic acid, amines, and precursors for the production of complex molecules.

#### Sabatier reaction

**Fig. 10:**
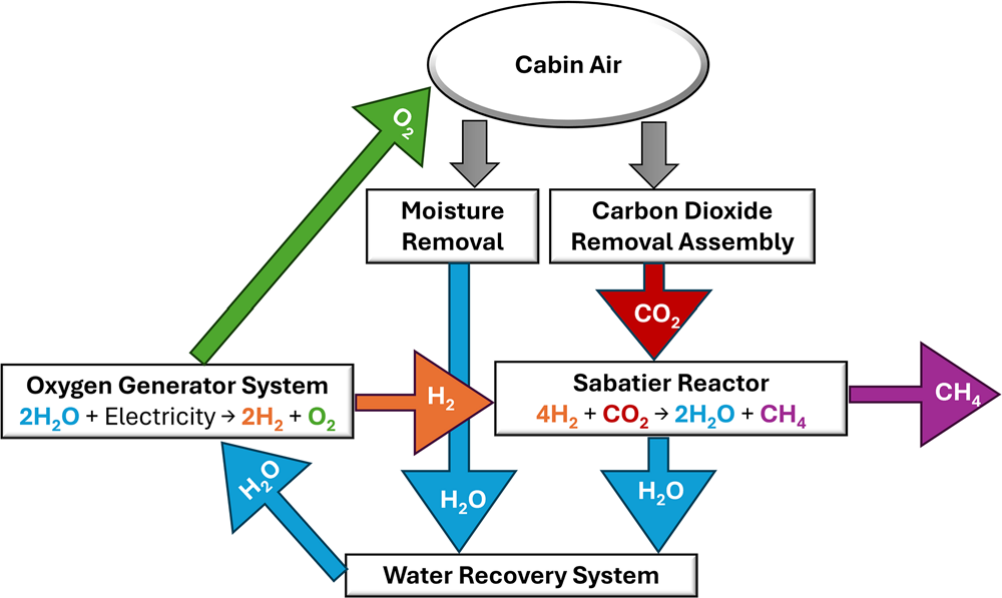
Diagram of Sabatier reactor within the ECLSS. Sabatier reactor integrated with oxygen regeneration, water recycling, moisture removal and carbon dioxide removal assembly.

Efficient use and recycling of both matter and energy are key to ensuring the sustainability of long-term space missions and any potential independent or semi-independent colony or station. The ISS is equipped with an ECLSS, which contains regenerative life support hardware that provides clean air and water to the ISS crew. The CDRA is a key component of this and processes over 4 kg/day of CO_2_ produced by the crew on the ISS^56^. Figure 10 outlines the conversions necessary to maintain efficient recycling of water and CO_2_, the oxygen generator system converting water into hydrogen and oxygen, and the Sabatier reactor converting hydrogen and carbon dioxide into water and methane.

**Fig. 11:**
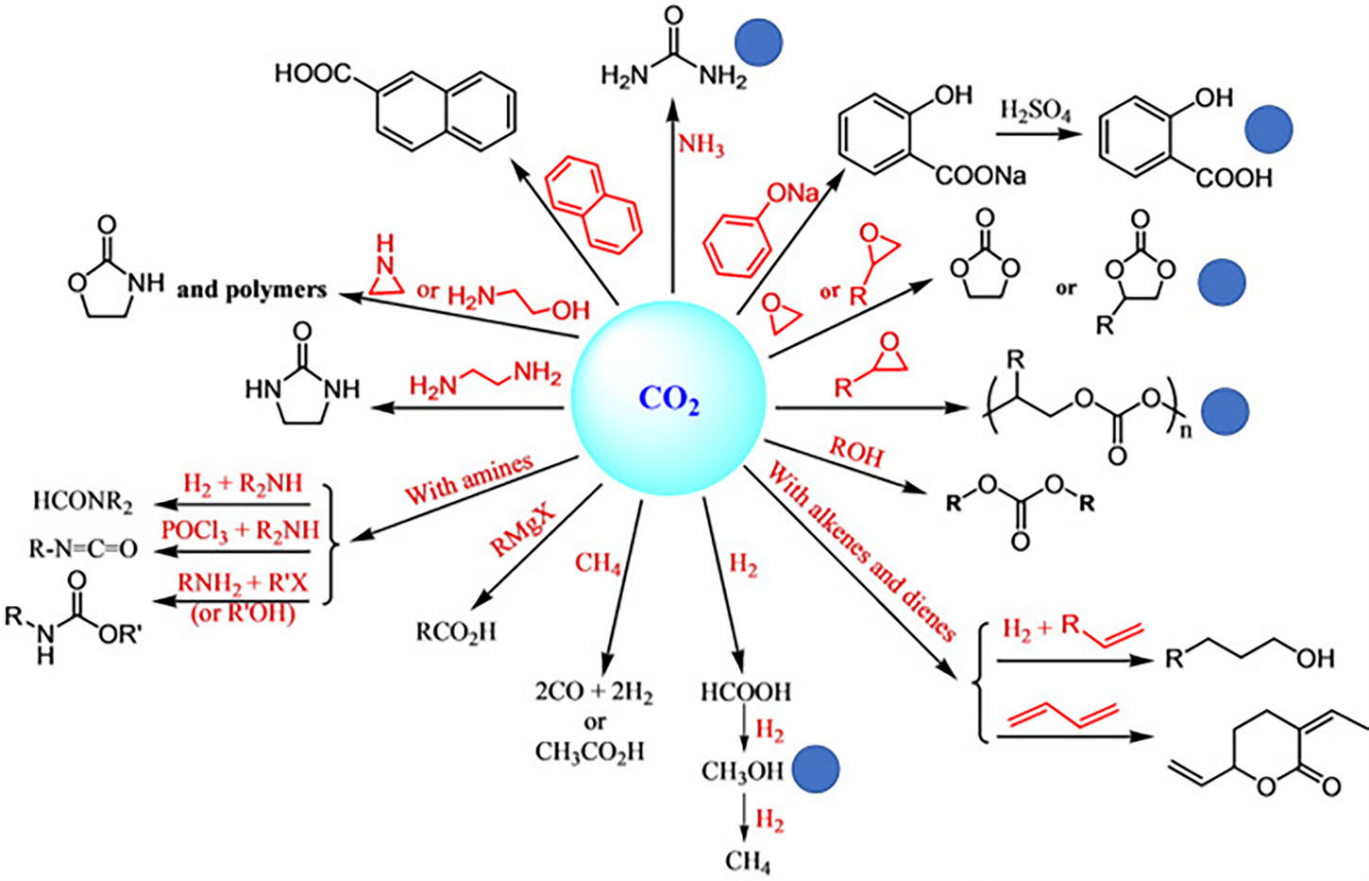
Schematic overview of CO_2_ conversion routes. Circles represent industrialized processes. Reproduced with permission from Tabanelli et al.^69^.

The ECLSS requires an estimated 9.6 kW of average power, which is 10–12% of the total power generated by the station’s solar arrays. The main part of the energy budget is used by the oxygen generation system at 4 kW, followed by the CDRA at 1 kW and then the water processor assembly at 0.3 kW^57^.

For a decade, the space shuttles provided water for the station as a by-product of the fuel cells they used to generate electricity. The Sabatier system supplemented the capability of resupply vehicles to provide water to the station, without becoming a sole source for this critical station resource^56,58^. In process mode, the Sabatier reactor operates at a temperature of 515 °C and ambient pressure; CO_2_ is entered the reactor at 48 °C to produce the desired products over a Hamilton Sundstrand proprietary ruthenium/alumina catalyst. The system recycled 50% of the CO_2_ produced on the ISS, and once initiated, was thermally self-sustaining with >99% efficiency for CO_2_ conversion^59^. Some concerns existed with system leaks, particulate formation and microgravity-induced separation that were discovered during testing. The methane produced by the Sabatier reaction was vented as it is considered hazardous to store flammable gas onboard the ISS. Eventually, the catalyst was poisoned, and the Sabatier reactor was shut down^3^. In the future, it is expected that liquefaction of methane could allow its storage and use as a propellant in the next generation of rocket engines, such as the SpaceX deep cryo-methylox engine currently under development. This is also attractive for refuelling on Mars.

#### Plasma pyrolysis

The methane produced from a Sabatier reactor could be converted to hydrogen and acetylene, recovering 75% of the hydrogen, as follows:

1 $$2{{C}{H}}_{4}\to {C}_{2}{H}_{2}+3{H}_{2}$$

The remaining hydrogen would be lost in the acetylene product that can be vented overboard. Wheeler et al. demonstrated this using the plasma pyrolysis assembly (PPA) under zero-g conditions upon a parabolic flight^60^. The PPA reactor contains an aluminium vacuum chamber connected to a microwave wave guide. A hydrogen-rich mixture of methane and hydrogen (4:1 ratio) is fed into the chamber while maintaining a pressure of approximately 110 torr. If pure methane is fed into the chamber, then higher hydrocarbons will be formed. The high-energy microwave ignites the hydrogen and methane into a plasma reaching up to 385 °C. The parabolic flight experiments confirmed the PPA’s ability to transition from an H_2_ plasma to a H_2_/CH_4_ plasma under zero-g, and ignite H_2_ plasma in Martian and Lunar gravities. This approach remains promising; however, specific challenges are noted, such as the handling of hazardous (explosive) gases aboard an aircraft.

#### Methane pyrolysis

Methane pyrolysis is the direct conversion of methane to hydrogen and carbon under high temperatures (>1000 °C). The hydrogen gas can be fed back to the Sabatier reactor, while the carbon-containing solid byproduct in the form of soot can be utilized in radiation shielding, electronics, and water purification, or sent to the waste management system. Other minor byproducts are also produced, including ethylene, acetylene and carbon monoxide. Childers et al. from Honeywell Aerospace developed a full-scale reactor and demonstrated 50-80% conversion with >95% selectivity^61^. Methane conversion increased with higher temperatures and decreased with higher flow rates (shorter residence times). Several factors could prevent the implementation of this technology on the ISS and other space habitats, including power consumption, size and weight constraints, maintenance requirements, durability and safety concerns. Practical improvements for future reactors include better material selection, simplified reactor opening/closing mechanisms, and enhanced substrate designs to maximize carbon deposition and minimize maintenance.

Methane pyrolysis can also be performed using the Honeywell carbon vapour deposition (HCVD) system^62^. Chemical (or carbon) vapour deposition is driven by methane decomposition on a surface. Carbon deposits on reactor surfaces or catalysts as a coating. Temperatures are similar or higher than traditional pyrolysis but optimized specifically for deposition kinetics. The carbon morphology is also different, where the deposition results in films, fibres, nanotubes or graphite layers, which can be utilized in a wider range of applications such as electronics or water purification^63^.

#### Bosch process

The Bosch process is considered an alternative approach to the Sabatier reaction for oxygen recovery. Instead of converting CO_2_ and H_2_ into water and methane, the Bosch process converts CO_2_ and H_2_ into water and solid carbon via two steps. The first step is via the reverse water-gas shift (RWGS) reaction, converting CO_2_ and H_2_ into carbon monoxide and water,

2 $${{C}{O}}_{2}+{H}_{2}\leftrightarrow CO+{H}_{2}O$$

The second step is the combination of two reactions occuring simultaneously, where CO and H_2_ are converted to solid carbon and water

3 $$CO+{H}_{2}\to C(s)+{H}_{2}O$$

and CO is converted to solid carbon and CO_2_ via the Boudouard reaction,

4 $$2CO\to C(s)+{{C}{O}}_{2}$$

The CO_2_ is recycled back into the process, creating a continuous closed-loop cycle, while the solid carbon can be utilized or disposed of. Theoretically, the Bosch process is capable of recovering 100% of oxygen from CO_2_, compared to the theoretical maximum of 50% recovery for the Sabatier reaction. However, the Bosch process is more complex, less reliable, difficult to package, creates solid carbon in undesirable locations, and requires more maintenance, according to a trade study by Abney et al.^62^. Overall, Sabatier was chosen over the Bosch process for the ISS because of its major engineering advantages, including smaller mass, lower power, lower volume, high technical maturity and lower development risk. However, Bosch becomes attractive for deep space missions where resupply of oxygen becomes extremely difficult or impossible. In these cases, the 100% oxygen recovery becomes critical.

Bosch can be utilized in different architectures, including Series-Bosch (SBOS) and Vertical Bosch (VBOS)^62^. SBOS separates the Bosch process into two main reactors, the RWGS reactor and the carbon formation reactor. This allows one reactor to operate while another undergoes carbon removal, where solid carbon is removed periodically. The architecture requires hydrogen extraction membranes, CO_2_ extraction membranes, a compressor and gas recycle loop, and a condensing heat exchanger to remove water. VBOS, on the other hand, implements the Bosch reaction within a single reactor configuration but uses multiple reactors in parallel. Parallel reactors provide redundancy if one reactor malfunctions or is inactive during carbon removal (required ~ 90 days).

Bosch can also be performed using ionic liquids, demonstrated via the ionic liquid carbon formation reactor (ILCF)^64^. The ionic liquid is used to regenerate the catalysts and remove the deposited carbon, hence addressing the issue of carbon fouling on catalysts.

#### Methanol synthesis

While the Sabatier reaction represents the main CO_2_ reduction pathway explored by NASA to date, there are a number of alternative CO_2_ reduction pathways that could be considered^65^:

- Reverse water gas shift
  5 $$C{O}_{2}+{H}_{2}\to {H}_{2}O+{CO}\,(\Delta {H}_{298}=+41\,kJ\,mo{l}^{-1})$$
- Methane synthesis
  6 $$C{O}_{2}+2{H}_{2}{\rm{O}}\to C{H}_{4}+2{O}_{2}$$
- Dry Reforming
  7 $$C{O}_{2}+C{H}_{4}\to 2{H}_{2}+2CO$$
- Sabatier reaction
  8 $$C{O}_{2}+4{H}_{2}\to C{H}_{4}+2{H}_{2}{\rm{O}}\,(\triangle {H}_{298}=-165\,kJ\,mo{l}^{-1})$$
- Methanol synthesis (two pathways)

9 $$C{O}_{2}+3{H}_{2}\to C{H}_{3}OH+{H}_{2}O\,\left(\triangle {H}_{298}=-49.1\,kJ\,mo{l}^{-1}\right)$$

10 $$C{O}_{2}+2{H}_{2}O\to C{H}_{3}OH+\frac{3}{2}{O}_{2}$$

The most promising in a space context is methanol synthesis, which, as proposed by George Olah, could form the basis of a future economic model in which methanol is the main source of energy^66^. Methanol has significant advantages in terms of safety, handling, storage and on-processing due to being a liquid under ambient conditions. As in the Sabatier reaction, methanol synthesis produces water as a by-product, which can be fed back into the water recovery system of the spacecraft. Terrestrial examples of CO_2_ to methanol recycling plants already exist in Iceland, by the Carbon Recycling International Company with an annual capacity of 3500 tonnes of methanol and in Japan, by Mitsui Chemicals with a 100 tonnes/pa demonstration plant, among others^67^.

#### Solid oxide electrolysis

Solid oxide electrolysis (SOXE) is a high-temperature electrochemical process used to convert carbon dioxide into oxygen and carbon monoxide. The system requires a cathode for CO_2_ reduction, an anode for O_2_ formation and an electrolyte for conducting oxide ions. The most common electrolyte material is yttria-stabilized zirconia. An impressive demonstration of SOXE was conducted on Mars aboard the Perseverance Rover, known as the Mars Oxygen In-situ Resource Utilization Experiment (MOXIE), conducted in 2020^68^. The oxygen could be used for breathing or to produce propellant, in combination with the carbon monoxide. MOXIE operation produced between 6 and 10 g of O_2_ per hour, roughly the breathing rate of a small dog. The SOXE technology required integration with critical sub-systems to enable operating in a Mars environment. For example, a scroll compressor was required to raise the pressure of the Martian atmosphere. Heaters and thermal insulation were required to maintain an operating temperature of around 800 °C. Overall, this is a promising approach, although scaling of 100–200 times would be required to support a human mission.

#### Other CO_2_ conversion routes

In addition to the reduction pathways given above, there are numerous synthetic routes for CO_2_ conversion whereby the entire CO_2_ moiety is incorporated into the product (e.g., carboxylation), see Fig. 11. There are advantages to this method in that the more direct synthetic procedure and greater increase in molecular complexity without intermediate platform molecules (e.g., MeOH or CH_4_) may lead to a reduction in overall energy consumption of the process.

One of the main drawbacks to utilizing CO_2_ as a chemical feedstock is its low energy value. To overcome this, a large energy input (usually in the form of heat) and/or the development of efficient catalyst systems is required. Four main strategies have been developed to overcome the thermodynamic stability of CO_2_^69^.

1. React CO_2_ with high-energy starting materials such as hydrogen, epoxides, or other small-membered ring compounds, unsaturated molecules, and organometallic compounds (e.g., Grignard’s reagents).
2. Choose highly oxidized low-energy molecules as synthetic targets, for example, organic carbonates or carbamates.
3. Use the Le Châtelier equilibrium principle, removing a particular compound to shift the equilibrium toward the product side.
4. Perform CO_2_ reduction under photoirradiation (UV light) or under electrolytic conditions.

#### Continuous flow conversion of CO_2_

This section outlines the state-of-the-art in CO_2_ conversion using low-energy methods (room temperature, photocatalytic, electrocatalytic) in continuous flow that may be suitable for adoption in a microgravity environment.

To date, there is very little precedent for synthetic chemistry in space. The main reason for this has been the inability of traditional chemical reactor technology to be compatible with a microgravity environment. The microgravity environment means that any headspace is problematic and has fundamental effects on convection and fluid dynamics. Akay et al. discuss the main obstacle of near-absent buoyancy in microgravity, where gas bubbles do not detach from the electrode surface^70^. In addition, there is difficulty in adapting such a platform to perform chemical transformations in a remote, automated fashion from Earth^71^. Flow chemistry can be used to overcome these fundamental issues whilst operating in self-contained modules with a smaller overall footprint and other advantages (see Table 2)^72^.

**Table 2:** Advantageous properties of flow reactors for space laboratories^72^

Property	Advantage in space
No headspace	Operation in microgravity
Closed reaction zone	Safety, no contamination
Precise control of parameters	Safety, reproducible
High-level automation and remote control	User-friendly
In-line analytical instruments	Compatibility
Wide range of chemical reactions	Advanced research
Often high conversion reactions	Less waste generation

A number of flow chemistry modules are already in use in space, including SpacePharma, Made In Space, Zaiput Flow Technologies, Beeler Research Group/Space Tango collaboration and in its own way, the ISSpresso Machine.

##### Sabatier reaction

**Fig. 12:**
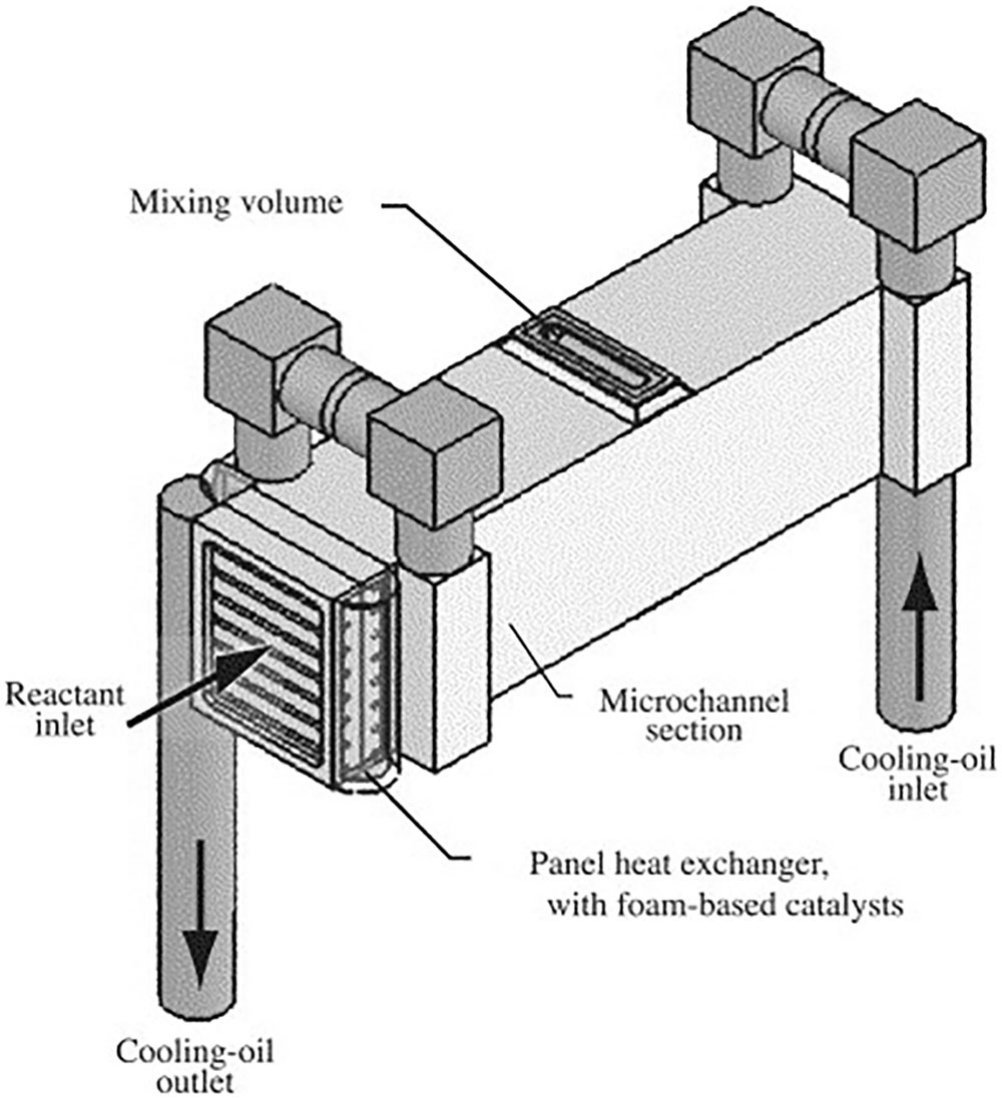
Engineering drawing of the Sabatier microchannel reactor assembly. Reproduced with permission from Brooks et al.^73^.

It has been demonstrated that the Sabatier process previously employed in the ISS can be translated to a microchannel reactor. This design offers the advantage of a compact reactor design with excellent thermal control (Fig. 12). By controlling the heat transfer along the length of the reactor, catalyst deactivation due to excess temperature can be avoided. In this approach, the reactive gases flow in rectangular channels that contain the Ru–TiO_2_ catalyst loaded onto a FeCrAlY metallic felt insert. The microchannel Sabatier reactor discussed in this paper is designed to deliver ~16 g/h of methane^73^.

Challenges exist with this thermal route, even using the efficient microchannel design. Selectivity of the desired products (H_2_O and CH_4_) is favoured at low temperature; however, because the reaction kinetics are slow at low temperature, it can be difficult to initiate the reaction. As such, the reaction is initiated and maintained at a temperature of 350 °C; removal of excess heat generated during the exothermic reaction can prove difficult at this already high temperature.

Many recent examples of the Sabatier reaction performed under continuous flow conditions have opted for a photocatalytic or photothermal approach. Here, CO_2_ methanation can be achieved at lower temperatures (<250 °C) using Ni-based catalysts^74,75^ as well as other transition metals (Co, Ru, Al, etc.) supported in different semi-conductors^76,77^. There is also potential for the use of solar illumination, either directly or as a means to power the required light source for photoreaction.

**Fig. 13:**
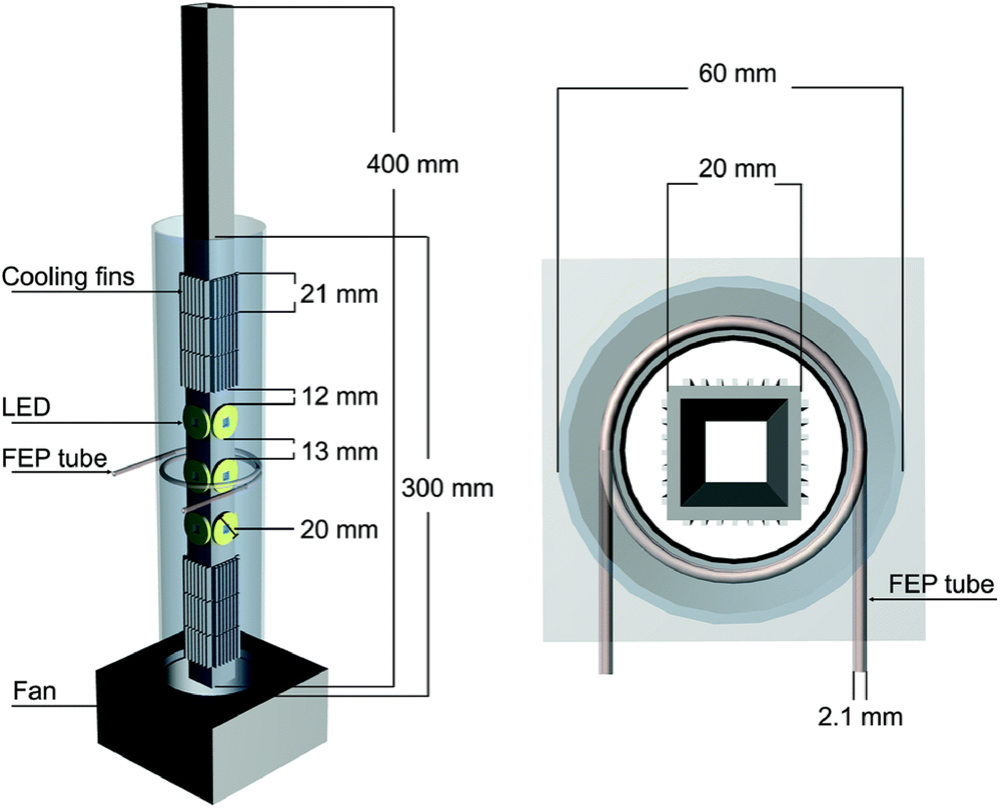
Diagram of the continuous photocatalytic reactor consisting of a UV-LED bar wrapped with FEP reactor tubing. Reproduced with permission from Dilla et al.^78^.

One common approach to continuous photoreactor construction has been to wrap a transparent, small-diameter tube around a light source to carry out reactions. In this example, a FEP tube with an inner diameter of 2.1 mm was illuminated by a self-made high-intensity UV-LED bar with a wavelength of 365 nm (Fig. 13). During illumination, the temperature of the reactor remained relatively stable at 26 °C. A fixed bed of TiO_2_ (P25) was placed in the FEP tube, held in place with glass wool. Both 14 and 26 cm reactor lengths were explored, with a peak CH_4_ formation of 3.6 nmol/h achieved in the 26 cm variant^78^.

##### Methanol synthesis

Methanol synthesis represents a compelling alternative to methane as a target for CO_2_ reduction via thermal, photothermal or photoelectrochemical methods. The thermal method involves physically heating the reagents using electric or gas heaters. The photothermal method converts light into heat to drive thermocatalytic reactions, while the photoelectrochemical method uses light to generate electrons, resulting in an electric field that drives electrochemical reduction. The thermal route typically involves catalytic hydrogenation at high temperature (300 °C) and pressure over Cu-ZnO catalysts. However, the competing reverse water gas shift reaction limits the overall yield of methanol produced. Recently, examples have emerged of photothermal routes to methanol, allowing for lower temperatures and pressures with high selectivity^79^. A microreactor design can allow for excellent mass and photon transfer due to the very high surface-area-to-volume ratio. One example of this approach is a sandwich-type design made from materials commonly used in photoelectrochemical and solar cells, with TiO_2_ thin films deposited on the FTO glass acting as the catalytic surface. A saturated solution of CO_2_ in 0.5 M NaOH was used as the reagent feed, which, after passing through the reactor at an optimum flow rate of 120 μL/min, gave 162 μM methanol in the product solution^80^.

**Fig. 14:**
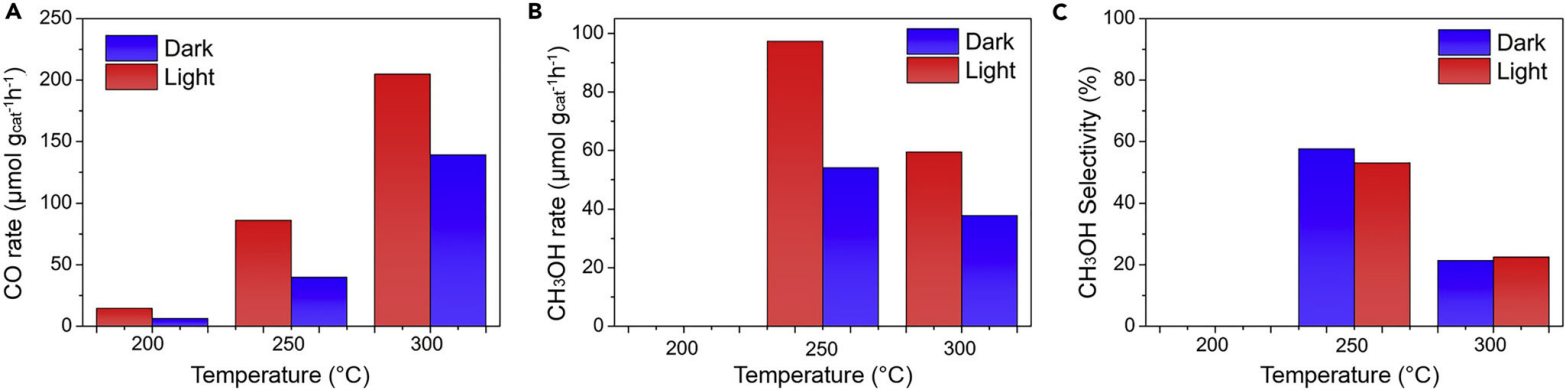
Methanol production rate and selectivity at different temperatures and atmospheric pressure. Carbon monoxide rate (**A**), methanol rate (**B**), and methanol selectivity (C) of the In_2_O_3−*x*_(OH)_*y*_ nanocrystal superstructure (NR-14h) in catalysing hydrogenation of CO_2_ with and without solar irradiation. Reproduced with permission from Wang et al.^81^.

Ozin et al. have demonstrated another approach with the use of defect-laden indium oxide, In_2_O_3−*x*_(OH)_*y*_, as an effective photothermal catalyst for the hydrogenation of CO_2_ to methanol with 50% selectivity at atmospheric pressure under simulated solar irradiation (Fig. 14)^81^. Production rates of up to 0.06 mmol g^−1^ h^−1^ were achieved, which is around 120 times higher than those of the best-known photocatalysts. The production rate remains two orders of magnitude lower than that achieved using thermally activated catalysts at 4.5 MPa and a similar temperature range^79^. However, the low quantum efficiency of the photothermal process (0.19% at 250 °C) means that there is considerable room for improving this process.

Although photoreduction of CO_2_ shows great potential, one of the greatest drawbacks is the low conversion efficiency. Herein, some of the key factors which limit the efficiency are listed: (1) mismatching between the absorption ability of semiconductor and the solar spectrum; (2) poor charge carrier separation efficiency; (3) low solubility of CO_2_ molecule in water (~33 μmol in 1 ml of water at 100 kPa and room temperature); (4) back reactions during reduction of CO_2_; and (5) competition reaction of water reduction to hydrogen^82^.

##### Electrochemical routes

**Fig. 15:**
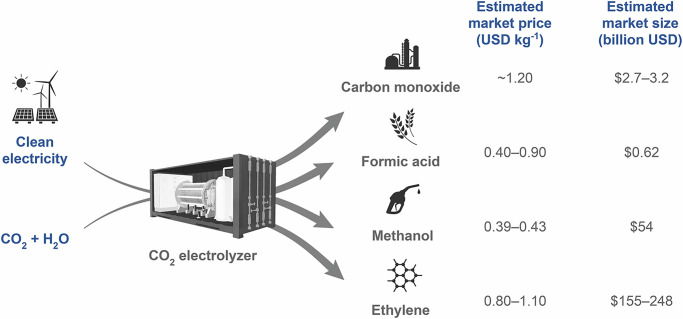
Overview of chemicals directly accessible via CO_2_ electrochemical reduction. Reproduced with permission from Weekes et al.^83^.

There are also many electrochemical (EC) approaches to CO_2_ conversion in which sunlight is first converted into electricity by a photovoltaic solar cell (PV) and then is used to electrochemically reduce CO_2_^70^. These electrolysers are capable of converting waste CO_2_ into useful fuel, chemical and polymer precursors or products (e.g., CO, methanol, ethylene, propanol, and others), as shown in Fig. 15^83^. Electric oxygen recovery (ELEC) was tested by NASA using a microfluidic electrochemical reactor (MFECR). The MFECR converted CO_2_ to ethylene (C_2_H_4_) using H_2_O as the proton source. This approach has a theoretical O_2_ recovery rate of 73%.

Electrochemical (EC) and photoelectrochemical (PEC) conversion of CO_2_ are multi-electron in nature (up to 8 e^‒^ for conversion to methane) with considerable kinetic barriers to electron transfer. It therefore requires the use of carefully designed electrode surfaces to accelerate electron transfer rates to levels that make practical sense, along with performing the reaction in a continuous-flow setup to overcome mass-transport limitations. Endrödi et al. discuss the most studied cell configurations, including the classical microfluidic setup, three different polymer electrolyte membrane (PEM) setups, and the solid-oxide electrolyser^84^.

One of the highest performing examples of a microfluidic CO_2_ electrolyser reports on a membrane-less electrolytic cell that implements pH-differential electrolyte configurations for converting gaseous CO_2_ into formic acid. The electroactive material was Pb using a 0.5 M K_2_SO_4_ (0.5 M H_2_SO_4_) electrolyte. All reactions were performed at room temperature and ambient pressure, achieving a current density of 345 mA cm^−2^ with formic acid formed as the main product with 95% Faradaic efficiency (the efficiency with which charge is transferred, facilitating CO_2_ reduction)^85^.

##### CO_2_ as a building block

**Fig. 16:**
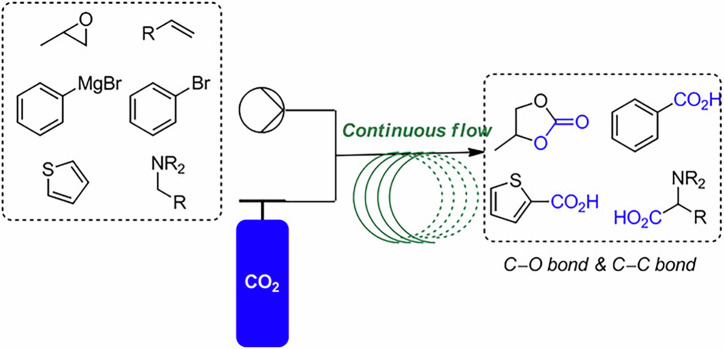
Carbon dioxide as a building block in continuous flow synthesis. Reproduced with permission from Sea et al.^86^.

As discussed in the Introduction, CO_2_ can be used as a building block in the formation of complex organic compounds. Continuous flow technologies have enabled C−O and C−C bond-forming reactions with CO_2_ that previously were either low-yielding or impossible in batch to afford value-added chemicals (Fig. 16)^86^.

##### C–O bond formation

The development of continuous flow methodologies for the fixation of carbon dioxide, in which specifically new carbon-oxygen bonds are formed, has been limited to the preparation of organic carbonates from epoxide precursors. Cyclic carbonates are broadly useful synthetic precursors and find important applications in the chemical synthesis of bioactive molecules; raw starting materials in plastics; electrolytes for lithium-ion batteries; and environmentally conscious ‘green’ solvents^86^.

**Fig. 17:**
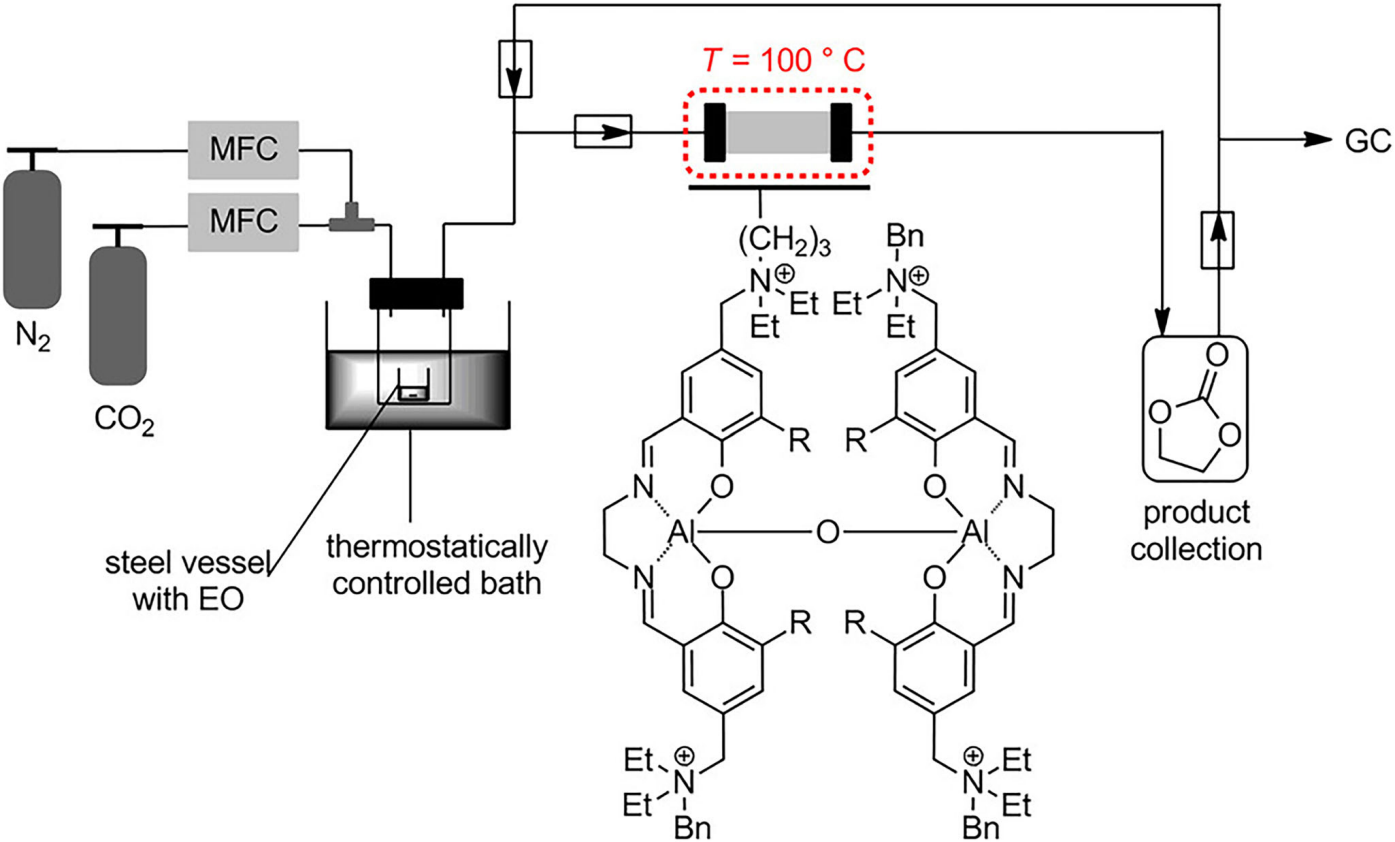
Continuous flow synthesis of ethylene carbonate from ethylene oxide and atmospheric CO_2_ employing an immobilized Al-salen complex. Reproduced with permission from Seo et al.^86^.

The first examples of the reaction of carbon dioxide with epoxides under flow conditions employed liquid supercritical carbon dioxide (scCO_2_) as both the solvent and C_1_ source with an immobilized catalyst in a fixed-bed reactor (Fig. 17)^87,88^. However, these processes require both high pressure and high temperature. The first use of atmospheric CO_2_ in the flow synthesis of cyclic carbonates was disclosed by North and co-workers in 2009^89^. Nitrogen gas and carbon dioxide were introduced into a steel vessel under cryogenic conditions containing ethylene oxide. The resulting gaseous ternary mixture was allowed to flow into a heated reactor column with an immobilized Al-salen catalyst (aluminium ions with salen ligands), enabling electrochemical conversion of ethylene oxide, CO_2_ and N_2_ to cyclic carbonates utilizing the C–O bond formation. Finally, a gas chromatography (GC) system with gas sampling valves for online monitoring of the system. The authors suggested that a hypothetical industrial-scale flow reactor could reach temperatures in excess of 150 °C in the absence of local waste heat, given the exothermic nature of EC formation (Δ*H*_T_ = −140 kJ mol^−1^). On this scale, an estimated 92,000 tons of carbon dioxide per annum could be removed and transformed into 184,000 tons of EC in a packed-bed flow reactor charged with 50 tons of immobilized catalyst.

A multicomponent flow system for the synthesis of cyclic carbonates from CO_2_ and olefins was developed by the Jamison group in 2014^90^. Employing atmospheric CO_2_ (introduced into the flow system by MFC), catalytic quantities (5 mol %) of either *N*-bromosuccinimide (NBS), benzoyl peroxide (BPO), and *N*,*N*-dimethylformamide (DMF) solutions of a variety of terminal epoxides at the mixer with residence times of 30 min at 120 °C (100 psi BPR), the corresponding cyclic carbonates were obtained in 49–89% yields.

##### C–N bond formation

**Fig. 18:**
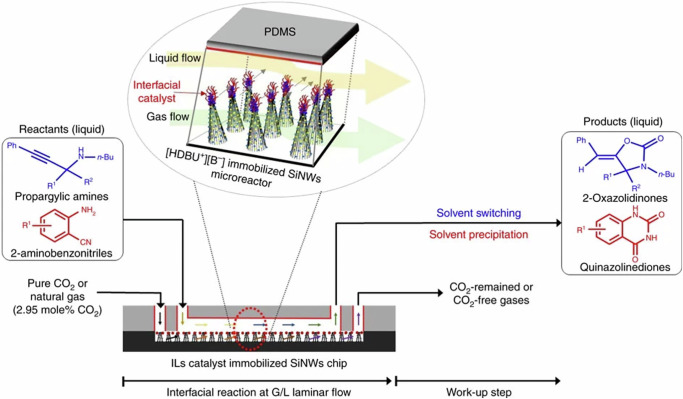
Microfluidic system for utilization of CO_2_ enabled by interfacial gas-liquid laminar flow catalysed by immobilized ionic liquids. IL = ionic liquids, SiNWs = silicon nanowires. Reproduced with permission from Vishwakarma et al.^91^.

In 2017, Kim and co-workers reported a catalytic system for capturing and fixing CO_2_ based on interfacial gas–liquid laminar flow catalysed by ionic liquids^91^. The microreactor system comprised upper and lower panels onto which highly amphiphobic silicon nanowires (SiNWs) were fixed. A liquid phase of an organoamine was flowed through the top panel while a gas flow containing CO_2_ was passed through the bottom panel; because of the high amphiphobicity of the supported SiNWs, a controlled gas-liquid interface was maintained throughout the channel. Catalysis was affected via a DBU-based ionic liquid that was immobilized on the tips of the SiNWs in the form of a ‘thimble’. The efficacy of the microfluidic system was demonstrated with two reactions conducted at the gas–liquid interface (Fig. 18): the reaction of CO_2_ with either propargylic amines or 2-aminobenzonitriles to prepare 2-oxazolidinones or quinazoline-2,4-(1*H*,3*H*)-diones, chemical motifs relevant to pharmaceutical applications.

**Fig. 19:**
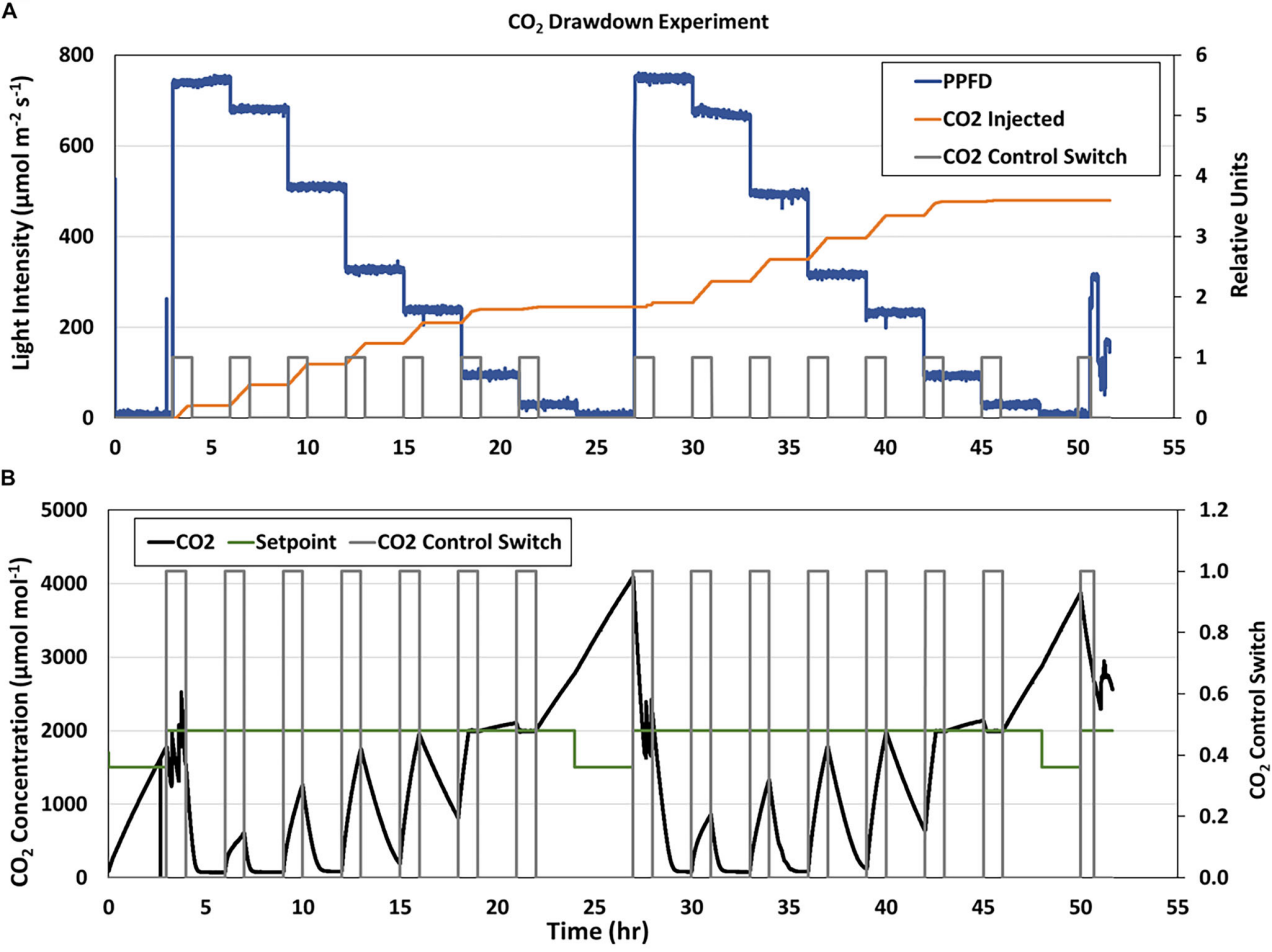
Biological conversion of CO_2_ by wheat on board the ISS. Light intensity, CO_2_ input (**A**) and CO_2_ drawdown by wheat (**B**) in sequential experiments in the APH on board ISS. The black line in (**B**) indicates the change in CO_2_ concentration in the APH under which wheat plants are photosynthesizing under the influence of decreasing light intensities. Reproduced with permission from Monje et al.^96^.

**Fig. 20:**
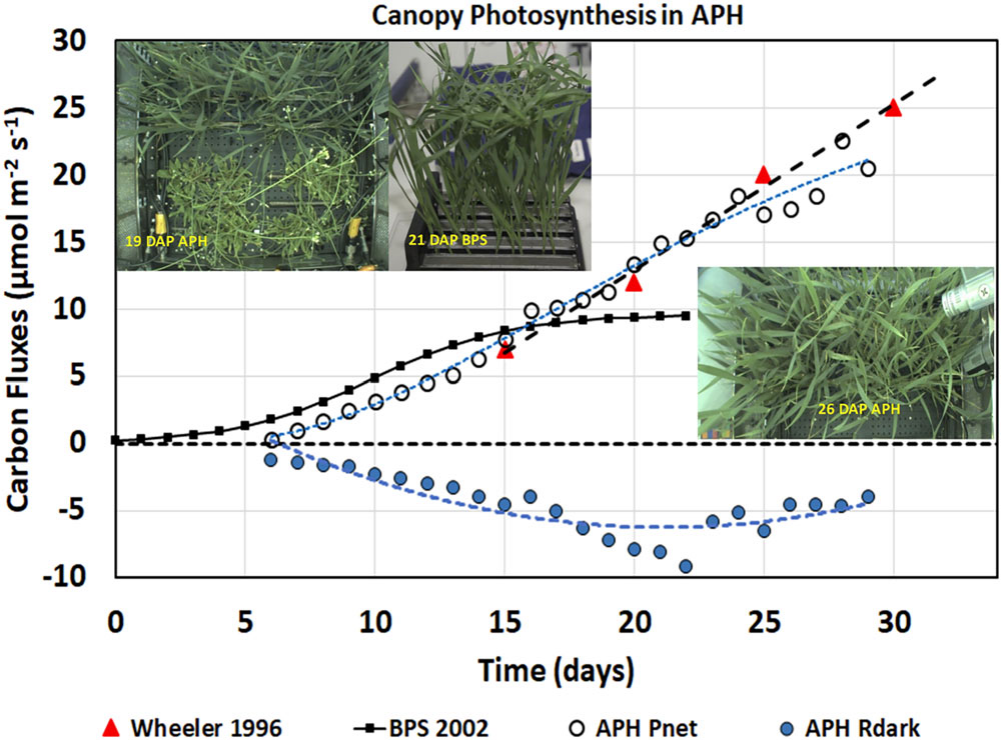
Daily net photosynthesis (*P*_net_) and dark respiration rates calculated from CO_2_ drawdown experiments by Monje et al. and a comparison with wheat plants grown on Earth. Reproduced with permission from Monje et al.^96^.

**Fig. 21:**
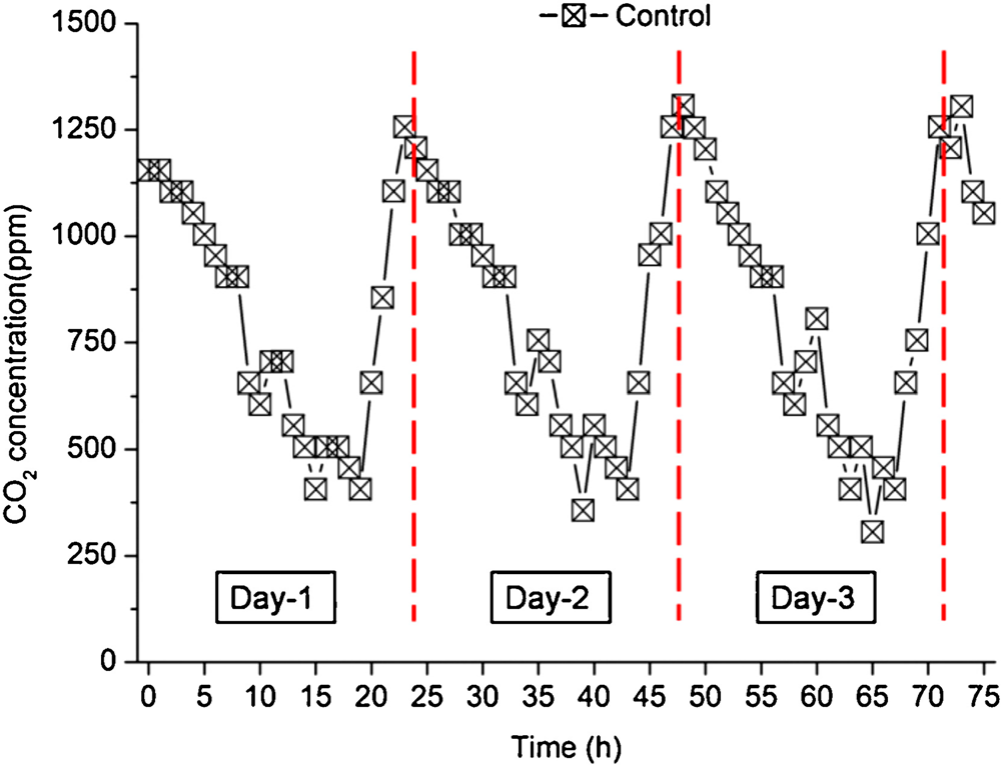
Diurnal CO_2_ fluctuations in the Lunar Palace during the steady state operation. Reproduced with permission from Dong et al.^99^.

This microfluidic approach utilizes the intrinsic advantages of high surface-to-volume ratio, efficient heat and mass transfer, and results in accelerated reaction kinetics under catalytic conditions and, more significantly, demonstrates the ability to employ CO_2_ as a building block for not only C−O bond formation but also for a tandem C − N bond forming event^86^.

##### C–C bond formation

Carbon–carbon bond-forming reactions with CO_2_ in continuous flow have emerged as a set of powerful, value-generating transformations. Traditional C−C bond-forming reactions affording carboxylic acid products usually employ stoichiometric amounts of strong nucleophiles such as organolithium/organomagnesium compounds. More recently, carboxylations based on the union of CO_2_ with less activated coupling partners have been reported under catalytic conditions.

In 2008, a continuous flow system for the carboxylation of pyrrole with scCO_2_ catalysed by an immobilized decarboxylase enzyme was reported by Matsuda and co-workers^92^. The decarboxylase underwent its reverse reaction under a high pressure of CO_2_ (984 psi) to afford pyrrole-2-carboxylic acid, demonstrating a yield improvement in the flow system of 25 times compared to the corresponding batch system.

The Jamison group reported a carboxylation of amines with CO_2_ via photoredox catalysis for the synthesis of α-amino acids in continuous flow in 2017^93^. This methodology included the inline generation of an α-amino radical and a CO_2_ radical anion followed by radical-radical combination by a photoredox catalytic cycle of *p*-terphenyl in the presence of potassium trifluoroacetate (KOCOCF_3_). This method demonstrated the first practical generation and use of the CO_2_ radical anion with the aid of a photochemical continuous flow system.

#### Additive manufacturing with CO_2_-based precursors

Long-term missions require a shift from transporting supplies from Earth to manufacturing in-space. If carbon dioxide can be converted to thermoplastics, then it is possible to manufacture spare parts and supplies on-board and on-demand. For example, Marchat et al. demonstrated the conversion of carbon dioxide into polyols that are used to make polyurethane found in mattresses and cushions^94^. As the In-Space Manufacturing (ISM) phrases it “Make it—Don’t take it”^95^. ISM consists of an integrated portfolio of on-demand production and recycling methods, including 3D printing in zero-gravity, additive manufacturing, multi-material fabrication with printed electronics, and refabricator technology.

3D printing is a promising manufacturing method for space due to its versatility and programmable nature. The method can also utilize waste plastic such as packing materials made of LDPE, HDOE, PET, Nylon and PVC. Fused deposition modelling (FDM) is a production method making use of a layer-by-layer thermoplastic polymer where there are no unbound loose powders and there is no solvent removal required.

Items that have been 3D printed include:

- A ratchet wrench was printed in 2014 on the ISS.
- Mini satellites (about 10 cm in size) called CubeSats are using PEEK.
- Artificial bones on the ISS by companies Made In Space and Allevi, using the first bio-printer in space.

### Biological conversion of carbon dioxide

#### CO_2_ studies on board the ISS

The conditions in orbit are known to affect plant physiology in several ways. Microgravity alters fluid dynamics, impacts liquid and gas exchange, changes capillary-driven moisture redistribution, leads to poor root zone aeration, and leads to loss of buoyancy-driven convection, causing sub-optimal mass and heat transfer to leaves^96^.

Monitoring and investigation of CO_2_ dynamics has been carried out to varying degrees in the various iterations of plant growth systems sent into orbit (Figs. 19–21). The growth systems on ISS are generally operated at elevated CO_2_ concentrations. In the early experiments growing lettuce in Veggie, the internal atmosphere of the bellows was exchanged with the cabin, keeping CO_2_ levels equal to the ISS cabin environment^97^. To date, this system has not been utilized for detailed studies of gas exchange or plant responses to elevated CO_2_ concentration.

The most detailed assessments of CO_2_ drawdown and photosynthesis rates carried out to date on the ISS were made in the Advanced Plant Habitat^96^. The APH is fitted with sensors to track both CO_2_ and water vapour. Validation studies of the unit included measurements of photosynthesis and plant respiration using the CO_2_ drawdown method, which also provides some insights into the potential impact of plants on the cabin atmosphere^96^. The larger size of the APH allowed for a larger plant species (wheat) to be grown, which tested both the root zone moisture control and the recovery of transpired water by the condensation system.

The tests confirmed that plants draw down the atmospheric CO_2_ concentration within the sealed chamber when photosynthesizing and releasing CO_2_ due to respiration during the dark phase.

A series of consecutive CO_2_ drawdown experiments at different light intensities (from 62 to 799 mmol/m^2^s at the plant surface) was carried out when the wheat plants were 24 days old. The results showed that 0.1 m^2^ of wheat plants in the APH were capable of rapidly decreasing the CO_2_ concentration in the chamber from 2000 mmol/mol to the set point of 1500 mmol/mol within 1 h at the light intensities above 533 mmol/m^2^ s. As light intensity decreased, the CO_2_ uptake rate slowed and, at the lowest light levels, even became negative, leading to increased CO_2_ concentration above the set point due to respiration. Monje et al.^96^ used the data from this experiment to calculate the canopy net photosynthetic rate (*P*_net_) and showed that both light intensity and CO_2_ concentration influenced the *P*_net_ of the wheat plants. Maximal *P*_net_ occurred at the highest light intensity and CO_2_ concentration used in this experiment, see Fig. 20.

These data were collected using non-destructive techniques with minimal intervention from the crew. The control of the growing system and the pre-programmed CO_2_ drawdown experiments were accomplished via teleoperation from the Kennedy Space Centre, demonstrating the utility of the remote command centre. Unfortunately, the experiment was not repeated on the ground, so it is not possible to use this data to draw any definitive conclusions about the performance of these plants in space^96^.

#### CO_2_ studies in earth-based simulation systems

Mass and energy balances have been carried out on several of the closed habitat simulations that have been built on Earth, which provide information about changes in atmospheric CO_2_ concentrations under the influence of both plants and human inhabitants.

One of the most recent and detailed experiments is the 105-day closed operation of the Chinese Lunar Palace^98^, see Fig. 21. During the experiment, three crew members lived in the closed habitat with fully recycled water, atmosphere and nutrient cycles. Sixty-nine m^2^ of hydroponics growing five grain crops and 15 different varieties of fruits and vegetables formed part of the water and atmospheric control system. The CO_2_ concentration varied from 500 to 5000 ppm with larger variation in the early stages of the experiment before a steady state concentration below 1000 ppm was reached after 60 days. The authors note that the ability of the 69 m^2^ of plants to drawdown CO_2_ produced by the crew varied depending on the occupancy. They found that the system was insufficient to meet the requirements for three male occupants (days 8–11) but was able to balance the CO_2_ produced by one male and two female occupants (days 26–105).

Dong, Liu et al.^99^, and Dong, Shao et al.^100^, reported a more detailed study on the plant response to elevated CO_2_ concentrations within the Lunar Palace during the 105-day occupation. They showed that, during the steady state period of operation, there was a strong diurnal cycle in CO_2_ production with CO_2_ concentrations decreasing when plants were photosynthesizing (lights on) and increasing when plants were respiring (lights off), see Fig. 21. They also showed that wheat plants responded to increased CO_2_ concentrations by increasing photosynthesis and reducing stomatal conductance up to a concentration of 1000 ppm CO_2_^100^. This led to increased biomass production and grain yields. Further increases in CO_2_ up to 5000 ppm were associated with decreases in plant performance (photosynthesis, biomass accumulation and grain yield) due to metabolic limitations. Higher CO_2_ concentrations were associated with higher water use efficiency as the reduced stomatal conductance decreases transpiration rates (releasing water back into the atmosphere). The elevated CO_2_ concentrations mean that CO_2_ uptake is not compromised by the reduced stomatal conductance; therefore, the amount of carbon assimilated per mL water transpired is increased under elevated CO_2_^100^.

The O_2_ concentration in the Lunar Palace was maintained in the range of 19.5% and 21.5% throughout the experiment. An average O_2_ production rate of 2093 g/m^2^/day was measured, which met the 1923 g O_2_/day required for human respiration. Wheat was the main staple crop in the Lunar Palace and also contributed approximately 33.32 g O_2_/m^2^/day. Leafy green crops (which are often targeted as desirable species for off-world plant growth systems) produced the lowest levels of O_2_ at 7.32 g/m^2^/day. This is an important consideration for closed habitats that are often not considered when assessing crop species for inclusion in these systems.

Concentrations of ammonia, carbon monoxide, formaldehyde, hydrogen sulfide, toluene, ethylbenzene, acrolein, ethanol, ozone, methane, sulfur dioxide, and nitrogen dioxide were measured at the beginning and end of the experiment and found to have remained below the spacecraft maximum allowable concentration (SMAC)^98^.

As well as achieving balanced O_2_ production, the Lunar Palace was able to close the loop on water recycling. All water used by the crew (potable water, bathing, laundry, and food preparation) and irrigation water for plants was recovered through condensation of transpired water vapour and treatment of urine and wastewater.

One gap in the system was the mass balance of food and solid wastes. A total of 1256 g/day of exogenous food and salts for the crew and minerals for plants had to be supplied to the Lunar Palace and a similar mass of solid residues was exported. Therefore, the system was only capable of achieving 55% of the food requirements of the crew (including 61% of plant foods). The overall closure of the system (water, atmosphere and solids) was calculated to be 97%^98^.

#### CO_2_ studies in other habitat simulations

Earlier studies have been carried out to measure CO_2_ drawdown during photosynthesis and generation during respiration in a number of previous iterations of closed Biological Life Support Systems, including the Biomass Production Chamber at Kennedy Space Center, the Laboratory Biosphere, Biosphere 2^101–103^. The Biosphere 2 experience was particularly enlightening. Reviews of this experiment revealed that one of the contributing factors to the failure to maintain a safe, breathable atmosphere in the sealed habitat was the consumption of O_2_ and generation of CO_2_ from microbial decomposition of the organic-rich rainforest soils that were imported as the growing substrate. The CO_2_ then interacted with the concrete in the structure to form CaCO3. This, along with a slow leak from the habitat, led to a decline in O_2_ concentration from 21% to 14% (low enough to cause health problems for occupants) over 16 months^104^. This highlights the importance of considering holistic CO_2_, O_2_ and H_2_O mass balances to ensure that atmospheric controls are stable enough to maintain a healthy environment in tightly closed systems

#### Mathematical models of CO_2_, O_2_ and H_2_O in ISS and gateway habitats

Putz et al.^105^ used data from NASA to model the impact of two small “pick and eat” growing systems on the cabin atmosphere of the ISS. They compared the impact of two VEGGIE units versus one growing unit, the size of an International Standard Payload Rack, in both a simulated ISS and a simulated lunar Gateway habitat using the MATLAB® based simulation tool V-HAB^105^. In both systems, they simulated the growth of lettuce and tomatoes using data gathered from the Baseline Values and Assumptions document from NASA to validate the model, including biomass production, O_2_ production, CO_2_ consumption and H_2_O transpiration rates. The model included the currently employed Carbon Dioxide Removal Assembly (CRDA), a physico-chemical unit that controls CO_2_ concentrations on the ISS, and considered the impact of plants growing alongside this unit.

Their model showed only slight influences of the growth of plants on cabin CO_2_ or O_2_ concentration when the production area was at the scale of the VEGGIE or ISPR^105^. They did, however, note that, even at this small production volume, the plants could have a significant impact on the cabin humidity and water recovered by the humidity control systems. They showed that a full ISPR of plants significantly increased the humidity and the load on the condensation system, but not outside the specified range of the ISS.

Zabel^106^ used a Modified Energy Cascade crop production model to simulate the behaviour of a closed cycle life support system during the start-up phase of the greenhouse. They included a phase prior to the arrival of the crew and following their arrival to investigate the significance of changes in system dynamics with the addition of human occupants. They also modelled a larger volume of plant than Putz et al. allowing for a greenhouse that produces 65% of the daily calorie intake of a crew of six in a closed system on the Moon or Mars.

His model showed that, prior to the arrival of the human crew, the system is unbalanced so the CO_2_ demand of the plants must be externally supplied (either imported from Earth or generated on site). If this requirement is met, then the system can generate considerable O_2_ that can fill the habitat prior to the arrival of the crew, greatly reducing the amount of O_2_ that needs to be transported to the habitat.

They also show that, after the arrival of the crew, there is value in staggering the sowing of crops to ensure that the uptake of CO_2_ and generation of O_2_ are not subject to spikes and depressions associated with harvest and re-sowing periods.

The MELiSSA project includes a component to develop a mechanistic mathematical plant model that allows investigation of the impact of off-world environmental conditions on plant morphology, physiology and biochemistry. Poulet et al.^107^ added a component to model the effects of microgravity and then used this model to explore the implications of reduced gravity, and the resulting decrease in free convection, on gas exchange at the leaf surface.

Their results showed that ventilation is an important factor in ensuring efficient gas exchange and that this has serious implications for plant biomass accumulation. Simulations of plants grown on Earth (gravity = 9.807 m/s²), with ventilation at 1 m/s or 0.5 m/s produced 2.5 times and 1.8 times more biomass, respectively, than plants with no forced ventilation^107^.

This effect was magnified under reduced gravity conditions. In simulations with Martian gravity (3.711 m/s²), 7% less biomass was produced than under Earth conditions with ventilation at 1 m/s.

In microgravity (10^-5^ m/s²) with ventilation at 1 m/s, 21% less biomass was produced compared to Earth conditions. As ventilation was decreased in decreased gravity environments, plant performance rapidly degraded. Plants grown in Martian conditions without ventilation produced 62% less biomass than those grown with 1 m/s ventilation. In microgravity, plants not provided with ventilation failed to grow. This modelling result reflects the results of experiments on the ISS, which showed that ventilation is a critical factor to support plant performance in low Earth orbit and shows that, as long as sufficient forced air flow is provided, it is possible to overcome the lack of convection in reduced gravity environments, allowing plants to grow at similar rates to 1 g conditions.

These models provide important testing grounds to select factors that need to be investigated experimentally, ideally in orbit, to improve the performance of closed systems.

#### Food and algae production systems

A number of plant growth systems have been developed and tested in orbit, particularly on the International Space Station (ISS). NASA’s Veggie system, deployed in 2014, was the first plant growth unit designed specifically for food production rather than purely biological experiments. The system uses LED lighting, a flexible growth chamber, and a passive nutrient delivery “root pillow” system to grow crops such as lettuce and leafy greens. Veggie experiments have demonstrated that plants grown in microgravity can be safe for human consumption and nutritionally comparable to Earth-grown plants. However, scaling plant growth systems for atmospheric regeneration presents challenges. Estimates suggest that replacing engineered CO₂ removal systems entirely with plant-based systems would require much larger growing areas and significantly more power, making them more suitable for future planetary habitats rather than current spacecraft^97,108,109^.

Veggie requires approximately 7 watts (W) of energy input to power the lights, fans and control system. The fan system is essential because a lack of convective flow in microgravity leads to problems with gas exchange at the leaf surface. The fans break the diffusion-controlled bubble of O_2_ and humidity that forms around the leaves and introduce CO_2_ to ensure that photosynthesis is not compromised. It is worth attempting to compare the power requirements of carbon dioxide removal from plant systems compared with the engineered CDRA. As noted earlier, CDRA requires around 1 kW and Veggie requires 7 W. If we make the following assumptions: Veggie produces 100 g of plant mass per experiment, 150 g of CO_2_ are removed per 100 g of plant mass, and 30 days per experiment. Then, a full-scale Veggie-like system designed to completely remove the equivalent amount of CO_2_ that CDRA removes would require 23 kW of power. Keeping in mind that the current Veggie design that powers lighting, fans and the control systems has not been optimized for this purpose. Therefore, a thorough redesign for power optimization should be conducted for a fair comparison. To understand the scale required, 5 large trees or 16 m^2^ of algae would be required to support one human. This means the approach is unsuited for the ISS, but could be considered for Lunar or Mars habitats.

An advanced successor to Veggie, the Advanced Plant Habitat (APH), provides improved environmental control, including adjustable light spectra, temperature regulation, gas monitoring, and automated imaging systems. Installed on the ISS in 2018, APH allows remote operation from Earth and has successfully supported full plant life cycles for species such as wheat and *Arabidopsis*^96,110^. Other orbital research facilities include the Japanese Plant Experiment Unit (PEU)^111,112^ and the European Modular Cultivation System (EMCS)^113–115^, which focus primarily on studying plant physiology in microgravity and variable gravity environments rather than crop production. Kiss et al. from the EMCS showed that phototropic bending, a process where a plant grows towards or away from the light source, becomes weaker when gravity increases from microgravity toward partial-g and 1-g. No direct effect on the amount of biomass produced is reported^114^.

In addition to spaceflight systems, several Earth-based simulation facilities have been developed to test large-scale BLSS concepts. The EDEN ISS greenhouse, located in Antarctica, demonstrated controlled-environment crop production in an isolated extreme environment and produced over 260 kg of fresh vegetables during a 10-month experiment^116–118^. This is equivalent to producing around 1 kg of biomass per day. The MELiSSA project, led by the European Space Agency, is developing a fully closed-loop ecological system that integrates microbial reactors, plant cultivation, and waste recycling to regenerate air, water, and food^119^. Similarly, China’s Lunar Palace facility has demonstrated a highly closed ecological system capable of supporting a crew for extended periods while recycling nutrients, water, and atmospheric gases^98,120,121^. Other prototype concepts, such as the Lunar Greenhouse, aim to develop lightweight inflatable cultivation systems suitable for lunar bases^122,123^.

Alongside higher plants, microalgae are considered promising organisms for life support systems due to their rapid growth rates, high photosynthetic efficiency, and fully edible biomass. Algae can convert CO₂ into oxygen and organic biomass while simultaneously recycling nutrients and helping purify water. Several microalgae species—including *Chlorella, Arthrospira* (*spirulina*), and *Chlamydomonas*—have been tested in spaceflight experiments. These organisms have demonstrated resilience to microgravity, radiation, and other spaceflight conditions, and inactive cultures can survive extreme environments such as vacuum exposure, improving system reliability during emergencies.

Experimental studies have shown that microalgae can effectively consume CO₂ and produce oxygen while generating nutrient-rich biomass suitable for human consumption or animal feed. In addition, microalgae can remove pollutants and heavy metals from wastewater and metabolize nutrients such as nitrogen and phosphorus, making them valuable for closing resource recycling loops in life support systems. Experiments on the ISS have also explored the production of valuable compounds such as astaxanthin, a powerful antioxidant produced by the microalga *Haematococcus pluvialis*^124–128^.

Overall, both plant cultivation and microalgae production represent promising biological approaches to sustaining human life during long-duration space missions. Plants provide food and psychological benefits while contributing to atmospheric regulation and water recycling, whereas microalgae offer efficient CO₂ removal, oxygen production, and nutrient recycling capabilities. Continued advances in controlled-environment agriculture, bioreactor design, and integrated life support systems will be necessary before these technologies can be deployed reliably in future lunar or Martian habitats.

#### Key challenges and research questions for space plant growth systems

Considered together, all of the studies discussed above highlight the potential for off-world plant growth systems to form part of an atmospheric control system by converting CO_2_ into biomass that can be fed to the crew to improve both their nutrition and their mental health. However, they also show that there are many fundamental and applied factors that require further study to ensure that biological life support systems are safe and reliable.

Carillo et al.^129^ provide an overview of the key challenges that scientists and engineers must overcome to grow plants in space. They note that the system must be able to operate within the constraints of limited resources and volume/space, microgravity, energy consumption, heat transfer and crew time. As pointed out in the previous section, the volume and power requirements for plant growth based on the Veggie system are currently prohibitive. It must also be able to withstand the elevated levels of cosmic radiation that bombard habitats in low Earth orbit and on the Moon and Mars. Research to select the most appropriate crops and then to optimize the light conditions for those crops is also needed. There is more research into the effect of microgravity on fluid mechanics and the impact this has on plant performance.

In addition to all of these factors, there are many research questions relevant to human nutrition and psychology, microbiology and microbiome which need to be addressed to optimize crew performance, particularly over extended missions.

## Conclusions

Recycling CO_2_ was found to be a real challenge for long-term missions that may prevent the establishment of a permanent human presence on the Moon and trips to Mars. Using the International Space Station (ISS) as a case study, the ISS receives cargo deliveries 8–9 times per year, including fuel, foods, clothing, medicine and other supplies. Considering the enormous delivery costs of 10,000–40,000 USD per pound, on-site production and recycling of every resource is critical for permanent presence in space. With a focus on CO_2_, this study has summarized methods to capture and convert CO_2_ into useful products, reducing the need for supplies from Earth.

Humans metabolically produce around 1 kg of CO_2_ and 2.3 kg of water per day, and four to six astronauts will inhabit the Lunar module or space station at any one time. This means that the confined environment of a habitat rapidly becomes toxic for the astronauts. Since 2022, NASA’s average 1-h limit for ppCO₂ in a nominal habitat is 0.4% (3 mmHg), while the general terrestrial 8-h exposure limit is 0.5% (3.8 mmHg), with humidity levels maintained between 40% and 60%. The CO₂ levels on the ISS have historically reached up to 5000 ppm (0.5%), exceeding these limits and contributing to reported health concerns such as headaches and impaired cognition. Most carbon removal technologies struggle to efficiently capture CO₂ at the low partial pressures required to meet these limits. Therefore, there is a continued need to improve CO₂ removal capabilities while also recycling the CO₂ into a range of products, including pharmaceuticals, foods and carbon-based materials.

An average of 7 tons of propellant is required each year to control the ISS in orbit. Even considering the lightest propellant (methane), one would need the breath of over 50 astronauts, assuming 100% efficiency, to produce such volumes. Fuels, therefore, were ruled out as a likely product; however, conversion technologies were considered when astronauts reach Mars and have access to an atmosphere of CO_2_. Between 2010 and 2018, O₂ was recovered from CO₂ on the ISS using a Sabatier reactor; this process resulted in a loss of elemental carbon and only recovered 50% of the O₂. The Sabatier catalyst was subsequently contaminated and operation ceased, meaning the captured CO₂ is currently vented overboard.

A study on the nutritional requirements of astronauts indicated that astronauts were not receiving the full range of nutrients, including a lack of potassium, calcium, vitamin D and vitamin K. Interestingly, there was an accelerated degradation of vitamins A, C, B1 and B6 in the space flight conditions. Indeed, there is an opportunity here to recycle CO_2_ into vitamins, considering the elemental compositions C_*x*_H_*x*_O_*x*_ and the small amounts required. Plant and microalgae capabilities could play a role here by engineering the organisms to produce higher concentrations of nutrients. Synthetic conversion routes using flow chemistry are another interesting alternative, or a combination of both synthetic and biological routes.

Over 40 medicines are stored in the ISS medicine cabinet. A study indicated that several of these medicines degraded at twice the rate of Earth's conditions, including ibuprofen, melatonin and sertraline. Considering that the success of a mission highly depends on the health of the astronauts, it is critical to have quality drugs on hand. Converting CO_2_ to certain medicines has been shown to be feasible and presents a promising method for CO_2_ recycling. Further work is required on the purification and isolation of products, as well as the miniaturization for confined spaces. Flow chemistry capability may play a role here to efficiently convert and separate products in micro-gravity.

Materials, clothing, tools and equipment were amongst many other potential products using CO_2_. There is a great interest in 3D printed materials, and a few tools have been 3D printed on the ISS as a demonstration. Polymers or polymer precursors can be produced from CO_2_ conversion technologies, including electrochemical routes. Conversion efficiencies and microgravity compatibility remain a challenge.

Carbon dioxide removal technologies have been used since the first space vehicles, such as Mercury in the 1960s. Conventional technologies included lithium hydroxide, zeolite molecular sieves and amine sorbents. Since then, many advances have been made, including tailored zeolites, metal oxides, ionic liquids and cryogenic separation. There remains the challenge of maintaining CO₂ levels below NASA’s current 0.4% limit, given the limitations of these technologies to capture CO₂ at low partial pressures.

Synthetic conversion of CO_2_ in space is a major challenge because most reactions require conventional batch reactors incompatible with micro-gravity environments. Flow chemistry capability can be used to overcome these issues whilst operating in self-contained modules with a smaller footprint. Benefits include no headspace in the reactor, closed reaction zones, precise control of parameters, high-level automation and remote control, in-line analytical instruments, a wide range of chemical reactions and high conversions. Flow chemistry-based platforms have been tested in space, including SpacePharma, Made In Space, Zaiput Flow Technologies, Beeler Research Group/Space Tango collaboration and ISSpresso Machine.

Many of the conversion routes require energy in the form of heat. Electrochemical and photoelectrochemical approaches have proven compatible with flow chemistry. Organic carbonates through carboxylation are among the possible products from CO_2_ conversion. They are found as materials for prosthetics, lubricants, fuel additives and precursors for polymers. Diphenyl carbonate, for example, is used in the production of high-performance polycarbonates, which are of great interest for space applications. Methane, methanol, formic acid and formaldehyde are also of interest. Production of amino acids was also reported using CO_2_ and amines as precursors.

Biological conversion of carbon dioxide using plants and microalgae was reviewed. Many platforms have been established, including on the ISS and Earth-based analogues. Over 200 experiments have been performed on the ISS. The main advantages include natural scrubbing of CO_2_ from the air, production of O_2_, removal of nutrients from wastewater, recycling of transpired water vapour and food production. Having access to fresh fruit and vegetables improves the physical well-being and morale of the crew. Challenges have included effects of micro-gravity on nutrient uptake, water delivery and growth development, time required by astronauts to maintain the plants, available space, effects of cosmic radiation, sub-optimal heat transfer to leaves and localized CO_2_ concentrations due to lack of gas exchange. Factors that have not been researched include human psychology, microbiology and microbiome.

EDEN ISS, a ground-based demonstration in Antarctica, was capable of producing around 1 kg of biomass per day. Micro-Ecological Life Support System Alternative (MELiSSA) is a biogenerative life support system using bio-geo-chemical cycles to fully recirculate gases, liquids and solid wastes. The Lunar Palace integrates plant growth with animal protein production using insects and was tested with 3 crew for 105 days. Most studies have operated in an environment comprising around 3000 ppm. High levels of CO_2_ up to 5000 ppm were shown to decrease the plant's performance due to metabolic limitations. Detailed studies have correlated the amount of CO_2_ removal with the light intensity to which plants are exposed. Mathematical models have been developed to explore the parameter space of biomass production, O_2_ production, CO_2_ consumption and H_2_O transpiration.

Microalgae are promising organisms due to their ability to produce complex organic compounds by using inorganic ions, CO_2_, water and light, and their ability to detoxify, decontaminate and remediate wastewater. Fifty-one algal experiments have been performed in space, including green algae, blue-green algae and euglenophyte. The experiments proved that microalgae could survive space conditions, including radiation, weightlessness, temperature fluctuations and even direct exposure to space vacuum. Antioxidants like astaxanthin were one of the valuable products produced by microalgae, which could serve as a dietary supplement for long-duration space exploration.

Overall, CO_2_ removal and recycling are critical capabilities for long-term crewed missions in space. State-of-the-art technologies can maintain breathable CO_2_ levels in confined environments; however, more research is needed in converting CO_2_ to useful products. Synthetic and biological approaches offer promising routes to convert CO_2_ to chemicals, foods, materials and medicines.

## Data Availability

No datasets were generated or analysed during the current study.
